# DNA Damage Regulates Translation through β-TRCP Targeting of CReP

**DOI:** 10.1371/journal.pgen.1005292

**Published:** 2015-06-19

**Authors:** Theresa B. Loveless, Benjamin R. Topacio, Ajay A. Vashisht, Shastyn Galaang, Katie M. Ulrich, Brian D. Young, James A. Wohlschlegel, David P. Toczyski

**Affiliations:** 1 Department of Biochemistry and Biophysics, Helen Diller Family Comprehensive Cancer Center, University of California, San Francisco, San Francisco, California, United States of America; 2 Department of Biological Chemistry, University of California, Los Angeles, Los Angeles, California, United States of America; The University of North Carolina at Chapel Hill, UNITED STATES

## Abstract

The Skp1-Cul1-F box complex (SCF) associates with any one of a number of F box proteins, which serve as substrate binding adaptors. The human F box protein βTRCP directs the conjugation of ubiquitin to a variety of substrate proteins, leading to the destruction of the substrate by the proteasome. To identify βTRCP substrates, we employed a recently-developed technique, called Ligase Trapping, wherein a ubiquitin ligase is fused to a ubiquitin-binding domain to “trap” ubiquitinated substrates. 88% of the candidate substrates that we examined were bona fide substrates, comprising twelve previously validated substrates, eleven new substrates and three false positives. One βTRCP substrate, CReP, is a Protein Phosphatase 1 (PP1) specificity subunit that targets the translation initiation factor eIF2α to promote the removal of a stress-induced inhibitory phosphorylation and increase cap-dependent translation. We found that CReP is targeted by βTRCP for degradation upon DNA damage. Using a stable CReP allele, we show that depletion of CReP is required for the full induction of eIF2α phosphorylation upon DNA damage, and contributes to keeping the levels of translation low as cells recover from DNA damage.

## Author Summary

Approximately 600 human genes encode enzymes that act as ubiquitin ligases, which facilitate the transfer of the small protein ubiquitin to thousands of substrate proteins; “tagging” with ubiquitin often promotes the degradation of the substrate by the proteasome. In this paper, we adapt a technique called Ligase Trapping for use in mammalian cells. Ligase Trapping is a highly accurate method for determining which substrates are targeted by a ubiquitin ligase. Here we use it to identify new substrates of the human cell cycle regulator βTRCP. Our screen was indeed highly accurate, as we were able to validate 88% of the candidate substrates we identified by mass spectrometry. Some of these new substrates were unstable proteins that were stabilized by inhibition of βTRCP, or of the entire class of ubiquitin ligases of which βTRCP is a part. However, others appear to be stable or redundantly-targeted substrates, which have been more difficult to identify with current techniques. This suggests that Ligase Trapping will be able to reliably identify new substrates of human ubiquitin ligases. Further, one of the new βTRCP substrates, CReP, is specifically depleted upon DNA damage, and depletion of CReP contributes to inactivation of the translational machinery upon DNA damage.

## Introduction

**Fig 1:**
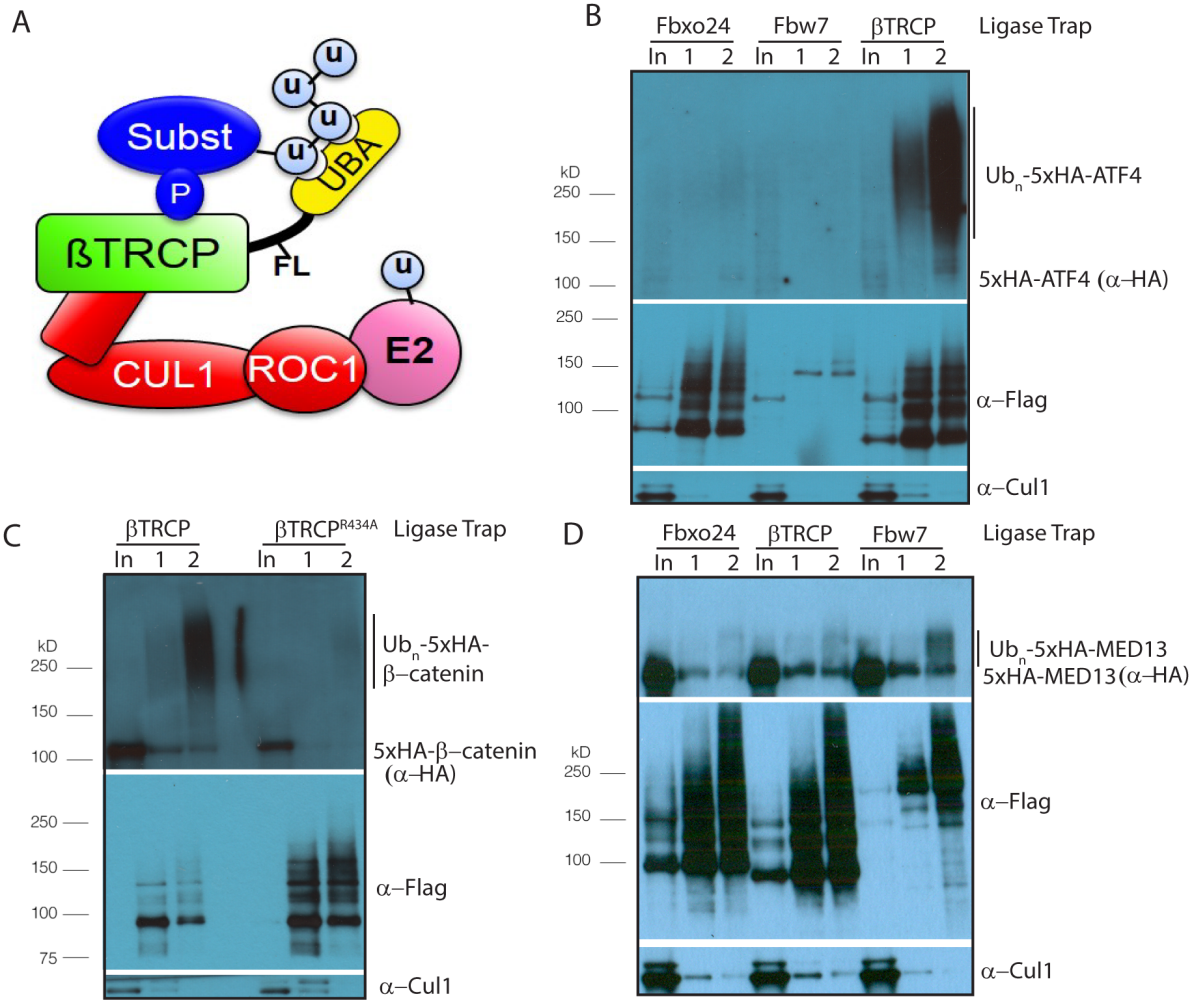
Establishing Ligase Trapping in human cells. (A) The SCF includes the scaffolds Skp1 (unlabeled, in red) and Cul1, which connect the E2-binding protein Roc1 to an F box protein such as βTRCP, which recruits substrates. Ligase Trapping is a two-step process in which ubiquitinated substrates are first precipitated under native conditions by a ubiquitin ligase fused to a UBA domain and then purified further under denaturing conditions via a 6xHis tag on ubiquitin. (B) βTRCP Ligase Trap purifies ubiquitinated species of the known substrate ATF4. Stable cell lines expressing the βTRCP Ligase Trap or a negative control (FBXO24 or Fbw7) were induced to express 6xHisUb for 3 days, transfected with 5xHA-tagged ATF4 for 24 hours, treated with 5 μM MG132 for 4 hours, lysed and subjected to a two-step precipitation. First, the Ligase Traps were purified under native conditions with anti-Flag antibody and eluted with Flag peptide. Then, the eluate was denatured in 6M urea and ubiquitinated proteins purified with NiNTA beads and eluted with imidazole. Loading was 1X input (In), 250X 1st step (1), and 5,000X 2nd step (2). (C) The interaction between the βTRCP Ligase Trap and the known substrate β-catenin depends on conserved substrate-binding regions in βTRCP. The pulldown in B was repeated, but without MG132 and with the substrate β-catenin as prey and both wt and mutant βTRCP as bait. (D) Fbw7 Ligase Trap specifically purifies ubiquitinated species of the known Fbw7 substrate MED13. Performed as in Fig 1B.

E3 ubiquitin ligases, which facilitate the attachment of anywhere from one to a long chain of the small protein ubiquitin to substrate proteins, are important regulators of the cell cycle and the response to stress. The best-studied outcome of ubiquitination is destruction of the substrate by the proteasome. There has been a great deal of interest in the discovery of ubiquitin ligase substrates, with the recent introduction of techniques that either look for proteins whose levels change when a particular ubiquitin ligase is inhibited [1–5], or those that use mass spectrometry to look for proteins that interact physically with the ubiquitin ligase [6–11]. Unfortunately, some ligase-substrate interactions are likely too weak to purify by affinity. Moreover, once a list of associated proteins is identified, it is not always clear which are direct substrates. To address this, most studies have determined whether the half-life of the substrate is significantly altered upon inhibition of the ligase [11]. However, in many instances, only a select fraction of substrate is targeted. In addition, some substrates are targeted redundantly by multiple ligases [12]. These facts often make it impossible to verify candidates merely by examining their half-life. For ubiquitin ligases for which a consensus binding sequence is known, the presence of this sequence has been used frequently to separate true substrates from non-substrate or non-specific interactors. However, this method is not useful to discover substrates of the vast majority of ubiquitin ligases, for which no consensus sequence is known. To eliminate these problems, we developed a technique called Ligase Trapping [13] (Fig 1A), in which an E3 ubiquitin ligase is fused to a ubiquitin-associated (UBA) domain. This mediates an extended interaction between the E3 ligase and its ubiquitinated substrates, allowing their co-immunoprecipitation. To distinguish between substrates and other associated proteins, this immunoprecipitate is subjected to a second purification for 6xHIS-ubiquitin under denaturing conditions. These purifications can be used both for substrate identification and as a diagnostic for candidate confirmation, in cases where the bulk level of a protein is stable.

The SCF is a cullin-RING ligase (CRL) containing 3 core catalytic subunits: the RING finger protein RBX1, the cullin CUL1 and the adaptor SKP1 [14–17]. This catalytic base associates with a substrate adaptor called an F box protein, of which humans encode at least 69. F box proteins are thought to recognize their substrates only after substrate modification, typically by phosphorylation [14,17]. Several of these F box proteins have been characterized due to their well-established roles as tumor suppressors and oncogenes. βTRCP[18] is an F box protein that turns over substrates to control the G2/M transition (*e*.*g*. WEE1 [19]/CDC25 [20,21]), as well as the response to DNA damage (*e*.*g*. CDC25 [20,21], claspin [7,22]).

In this paper, we establish ubiquitin ligase trapping in mammalian cells. Of the 28 candidates identified using this technique, 12 were well-established substrates [6,20,21,23–33]. For the 16 remaining candidates, we examined 14 and found that 11 of these confirmed. Thus, 23 of the 26 known/tested candidates, (88%) appear to be substrates, suggesting that Ligase Trapping is a robust discovery technique. Further characterization showed that turnover of one of the βTRCP substrates, CReP, is exacerbated by DNA damage. CReP is a protein phosphatase 1 (PP1) specificity subunit that counteracts the phosphorylation of eukaryotic initiation factor 2 alpha (eIF2α) on serine-51 [34], a stress-induced modification that inhibits translation initiation on most transcripts [35,36]. Inhibiting the turnover of CReP after DNA damage significantly reduced the accumulation of serine-51 phosphorylated eIF2α, and increased translation after DNA damage, suggesting that CReP turnover is an important mechanism by which DNA damage regulates translation.

## Results

To establish Ligase Trapping in human cells, we created a stable HEK293 line in which 6xHIS-ubiquitin is expressed upon treatment with doxycycline. In this cell line, tagged ubiquitin accounts for a significant portion of the total ubiquitin pool when cells are treated with doxycycline (S1A Fig). In yeast, fusion of F box proteins, via a 3xFlag linker, to the UBA of Dsk2 or the two tandem UBAs of Rad23, led to enhanced purification of nascent ubiquitinated F box protein substrates [13]. We fused the human F box protein βTRCP to the human homologs of these UBA-containing proteins, and found that the RAD23B fusion increased the poly-ubiquitinated species purified by the βTRCP fusion most strongly (S1B Fig). Accordingly, we made a stable cell line that expressed both doxycycline-inducible 6xHIS-Ub and a Ligase Trap consisting of βTRCP fused on its C-terminus to 3xFlag and the C-terminal UBAs of RAD23B.

To determine whether the βTRCP trap was functional, we expressed an epitope-tagged allele of the βTRCP substrate ATF4 in our stable cell line. We were able to immunoprecipitate poly-ubiquitinated ATF4 with the βTRCP trap, but not with the Ligase Traps of two unrelated F box proteins, FBXO24 and Fbw7 (Fig 1B). We obtained a similar result with β-catenin (S2 Fig). We also purified ubiquitinated forms of the Ligase Traps, which was unsurprising as many ubiquitin ligases are themselves ubiquitinated. We also purified substantial unmodified forms of the Ligase Traps. This is likely a result of the very large amount of IP loaded relative to input (5,000:1 for the 2^nd^ step), which is necessary to see the very small percentage of substrate that is poly-ubiquitinated. Even in cases where the unmodified band is equal in intensity in the input and 2^nd^ step IP, this represents only 0.02% IP background. This phenomenon also occurs frequently with unmodified substrates, while the relevant purification of poly-ubiquitinated substrates is highly specific to the relevant Ligase Trap. To examine further whether the purification of β-catenin was specific, we made a stable cell line identical to our βTRCP ligase trap line, but with a mutation in the WD40 domain of βTRCP predicted to prevent binding to β-catenin [37]. As expected, this mutant trap failed to purify polyubiquitinated β-catenin (Fig 1C), showing that β-catenin purification by βTRCP represents a specific interaction. To make certain that the βTRCP Ligase Trap didn’t simply bind all ubiquitinated proteins more efficiently, we made a similar stable cell line expressing Fbw7-3xFlag-RAD23. Poly-ubiquitinated forms of the known Fbw7 substrate MED13 [10] were preferentially precipitated with the Fbw7 Ligase Trap (Fig 1D).

Having established the functionality of the βTRCP ligase trap cell line, we performed a large-scale, two-step purification and identified ubiquitinated co-precipitating proteins by mass spectrometry. Before collection, we treated cells with the proteasome inhibitor MG132 for four hours, as we had shown that this treatment increases the amount of poly-ubiquitinated material purified by the βTRCP ligase trap (S1C Fig). We defined candidate βTRCP substrates as those proteins identified in at least two of three purifications of the βTRCP ligase trap, but not in any of the negative control purifications. Twenty-eight proteins met these criteria (Table 1). Of these, twelve were previously-validated βTRCP substrates, and many others had been shown to interact with βTRCP in previously published large data sets, but had not been individually examined to determine if they were substrates [4,8,11,38–40]. SUN2 was purified in a large-scale screen for βTRCP substrates, and shown to be stabilized by the proteasome inhibitor MG132 [39] while this manuscript was under review. In addition, several other known βTRCP substrates, such as ß-catenin [41–45], were selectively identified in the βTRCP purification, but as some peptides were also identified in control purifications, these did not meet the stringent criteria that we had chosen for this initial analysis (bottom of Table 1). The large fraction of previously-published substrates (43%) that we purified confirms that Ligase Trapping accurately identified true substrates.

**Table 1:** Discovery and validation summary for identified βTRCP substrates. List of all proteins purified uniquely and at least twice by the βTRCP Ligase Trap, with total spectral counts (TSC) for each of three purifications. Two unique HLA alleles were excluded, as other HLA alleles were identified in negative control purifications. Substrates in normal text were previously well-described, and those in bold are novel, although some have previously been isolated in large-scale experiments. The substrates below the black bar are known βTRCP substrates that were isolated, but did not meet our criteria for candidates. For the validation experiments, a blank box means the experiment was not performed.

Candidate Substrate	Locus ID	TSC1	TSC2	TSC3	Ubiquitinated forms precipitated?	Stabilized by beta-TRCP knockdown?	Stabilized by MLN4924?	beta-TRCP consensus binding motif
**HIVEP1/2**	P15822/P31629	13	0	69	yes		*	DSGESEEE
Nrf2	Q16236	18	8	9				
**CReP**	Q5SWA1	12	11	13	yes	partial	yes	DDGFDSD
**UBE4B**	O95155	17	5	23	yes	stable	stable	DTTFLLD
ATF-4	P18848	11	9	19				
CDC25A	P30304	11	7	14				
**ZNF395**	Q9H8N7	9	6	11	yes		yes	DSGSSTTS
**ZNF704**	Q6ZNC4	7	3	9	yes	partial	partial	DDGIDEAE/SDGEED
PDCD4	Q53EL6	5	4	4				
bHLHE40	O14503	5	2	14	yes		*	
CDC25B	P30305	3	2	10				
**BAT2**	P48634	2	3	6	no			DSGGSSSE/DSGVDLS/DSGHCVPE
Deptor	Q8TB45	3	2	7				
**SUN2**	Q9UH99	3	3	0	yes		yes	DDGSSSS
**AEBP2**	Q6ZN18	1	2	6	yes	partial	partial	SDGEPLS
RAPGEF2	Q9Y4G8	3	0	5				
**GGNBP2**	Q9H3C7	2	1	3				DSGKGAKS
TFAP4	Q01664	3	0	3	yes		yes	
Emi1	Q9UKT4	1	2	0				
Per2	O15055	2	0	3				
**ALDH2**	P05091	1	0	6	no			DGDFFSYT
WWTR1	Q9GZV5	2	0	2				
**TRIM9**	Q9C026	1	1	2	yes		*	DSGYGS
**CEP44**	Q9C0F1	1	1	0	no			SSGKSE
**DACT1**	Q9NYF0	1	1	0				SSGFYELS
**FNIP1**	Q8TF40	1	1	0	yes		no	DSGIARS
**RIPK4**	P57078	1	0	3	yes			DSGAS
**RASSF3**	Q86WH2	0	1	2	yes	no	no	SSGYSS
*NFκB p100*	Q00653	1	0	11				
*β-catenin*	P35222	33	18	36				
*eEF2K*	O00418	0	0	6				
*REST*	Q13127	0	0	12				

* indicates that the protein appears stable even in the absence of the cullin inhibitor MLN4924. The closest to consensus βTRCP degron found in the primary sequence of each novel candidate is shown.

We also purified substrates of Fbw7 using a Ligase Trap. The Fbw7 Ligase Trap was expressed at a low level, suggesting that this trap was less stable. However, the proteins pulled down most abundantly and specifically by the Fbw7 Ligase Trap were MED13 and MED13L, two members of the Mediator complex shown to be Fbw7 substrates in a recent screen[10] in which Fbw7 interactors were precipitated and identified by mass spectrometry. (Our purification of MED13 is shown in Fig 1D) In that screen, the entire 26-member Mediator complex was purified, and MED13 and MED13L had to be identified as the direct Fbw7 substrates by a combination of degron prediction and careful validation; we did not purify any other members of the Mediator complex.

**Fig 2:**
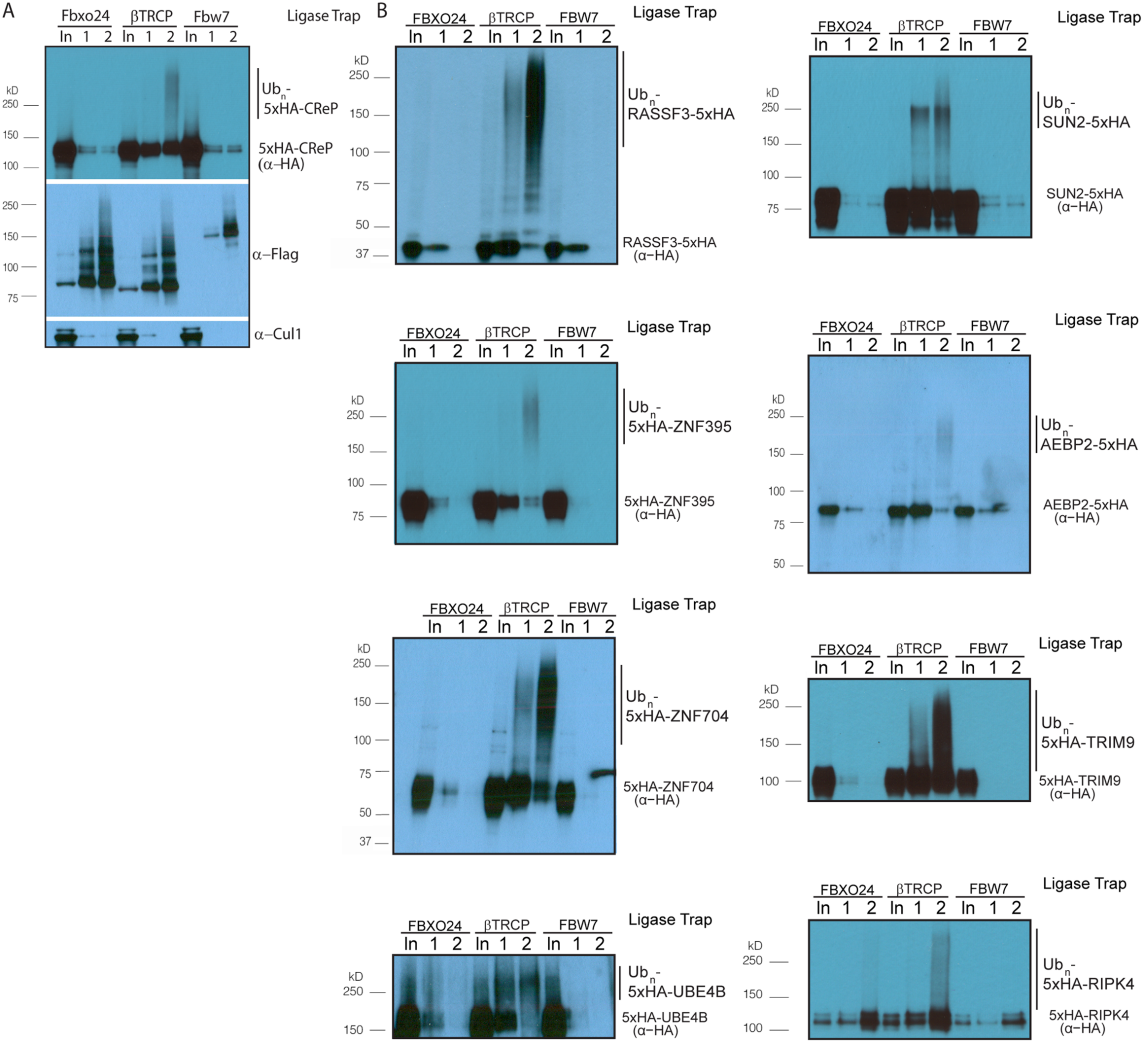
Validation of novel βTRCP substrates. (A) βTRCP Ligase Trap specifically purifies ubiquitinated species of the novel βTRCP substrate CReP. Performed as in Fig 1, without MG132 treatment. Loading was 1X for input, 250X for the 1^st^ step, and 5,000X for the 2^nd^ step. (B) Validation of additional candidate substrates. Loading controls and the rest of the substrates are in S3 and S4 Figs.

Ligase Trapping also provided a method to validate candidates beyond simply examining substrate turnover. Ligase Trapping is able to show that a ubiquitinated substrate specifically purifies with a particular ligase even if the substrate is redundantly targeted by multiple ligases, or if only a small fraction of the substrate (such as that in a particular complex) is ubiquitinated. To fully assay the accuracy of the Ligase Trap technique, we decided to validate candidate βTRCP substrates. Out of fourteen of the previously unknown/unvalidated candidates that we examined, eleven showed specific purification of polyubiquitinated material by the βTRCP ligase trap (Table 1 and Figs 2, S4 and S5). This strongly suggested that these candidates are true substrates of βTRCP, and that this technique accurately identified substrates with low background and thus will be an efficient way of identifying and validating substrates of other ubiquitin ligases in the future. Two βTRCP candidate substrates were not examined due to technical difficulties.

**Fig 3:**
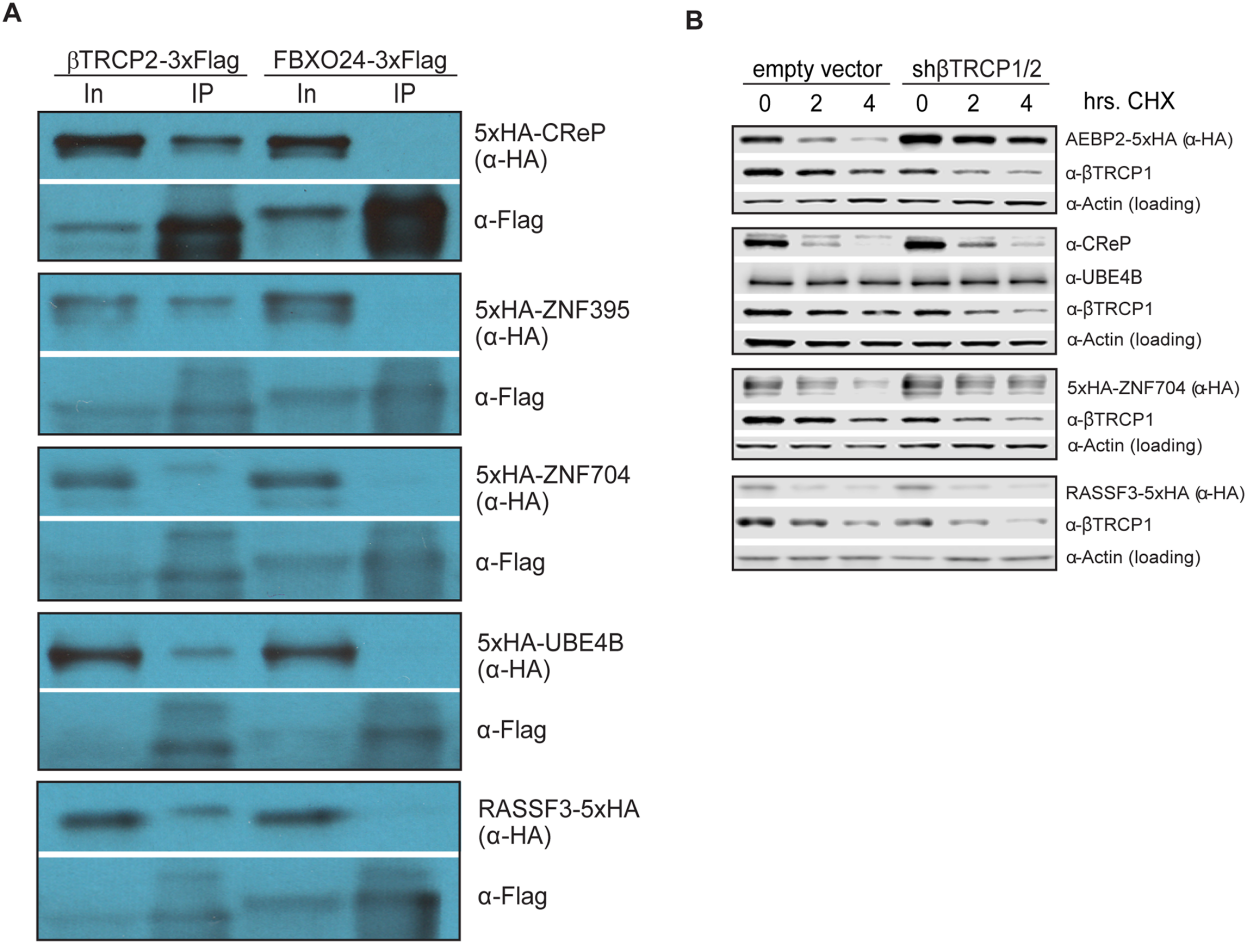
Ubiquitin ligase binding and turnover of a subset of novel βTRCP substrates. (A) βTRCP binds to its candidate substrates *in vivo*. HEK293 cells were transfected with 3xFlag-tagged F box proteins and 5xHA-tagged substrates for 1 day, lysed and subjected to a one-step precipitation. The F box proteins were purified under native conditions with anti-Flag antibody and eluted with Flag peptide. Loading was 1X input (In) and 75.3X IP for CReP, and 1X input (In) and 83.7X IP for other substrates. (B) Effect of βTRCP knockdown on candidate substrate half-life. HEK293 cells were co-transfected with a negative control plasmid, or a plasmid encoding an shRNA targeting βTRCP1 and 2, and a plasmid encoding a tagged βTRCP candidate substrate. Cells were treated with 100 μg/mL cycloheximide for the indicated time before collection.

In order to determine whether βTRCP could bind its candidate substrates in the absence of the UBA domains present in the Ligase Traps, we co-expressed Flag-tagged versions of these F box proteins in HEK293 cells with HA-tagged versions of a subset of their candidate substrates. In all cases, the substrate was purified more efficiently by its cognate ligase than by the negative control ligase (Fig 3A).

Because a common outcome of ubiquitination by the SCF is proteasomal degradation of the ubiquitinated protein, we assayed whether a subset of the candidate substrates were degraded in a way that depended on the cognate ligase. For five of the βTRCP candidate substrates, we co-transfected cells with DNA encoding tagged substrate, as well as a negative control plasmid or a plasmid expressing an shRNA targeting both paralogs of βTRCP, then inhibiting bulk protein translation with cycloheximide and assaying substrate levels. Although the knockdown we achieved was quite modest, three of the five substrates were significantly stabilized (Fig 3B). One, RASSF3, was not stabilized, suggesting either that it is a better βTRCP substrate than the others, or that it is targeted by other ubiquitin ligases. UBE4B is a stable protein. (Note that we detected UBE4B with a specific antibody against this protein, and did not ectopically express it, so its stability is unlikely to be an artifact.) It is possible that either only a small pool of the substrate was targeted, or that the outcome of ubiquitination of UBE4B is not proteasomal degradation.

Several commonly-used approaches identify ubiquitin ligase substrates as those proteins whose abundance is increased by inhibition of the relevant ligase. One key advantage of ligase trapping is that, in contrast to these techniques, it can identify substrates whose bulk turnover is not affected by inhibition of the ligase. To determine more universally which substrates were quantitatively targeted for degradation by βTRCP, we expressed tagged versions of the substrates, inhibited protein synthesis with cycloheximide, and followed the turnover of the substrate in the absence or presence of MLN4924 (Table 1 and S6 Fig). Of the ten substrates examined, three (CReP, ZNF395, and SUN2) were unstable proteins that were stabilized by MLN4924, suggesting that their turnover is mediated by βTRCP alone or in combination with other cullin-RING ligases. (CReP was previously shown to be an unstable protein [34], as was SUN2.) Four (ZNF704, FNIP, RASSF3 and AEBP2) were not or only partially stabilized by MLN4924, suggesting that these might be redundantly targeted by βTRCP and a non-CRL ligase. Three proteins (HIVEP2, UBE4B, and TRIM9) appeared to be constitutively stable, although we cannot rule out that overexpression or epitope tagging of HIVEP2 and TRIM9 led to an artifactual stabilization. βTRCP could be promoting non-degradative ubiquitination of these substrates, or may only ubiquitinate a specific pool.

We were initially concerned that treating cells with MG132 would lead to increased background, or skewing of the results. Therefore, we performed two purifications of the βTRCP ligase trap in the absence of MG132. This purification generated a list with several of the same substrates, but lacking a subset, especially those shown to be unstable in Figs 3B and S6 (S7 Fig). In addition, all of our validations were performed in the absence of MG132 (Figs 2, S4 and S5).

**Fig 4:**
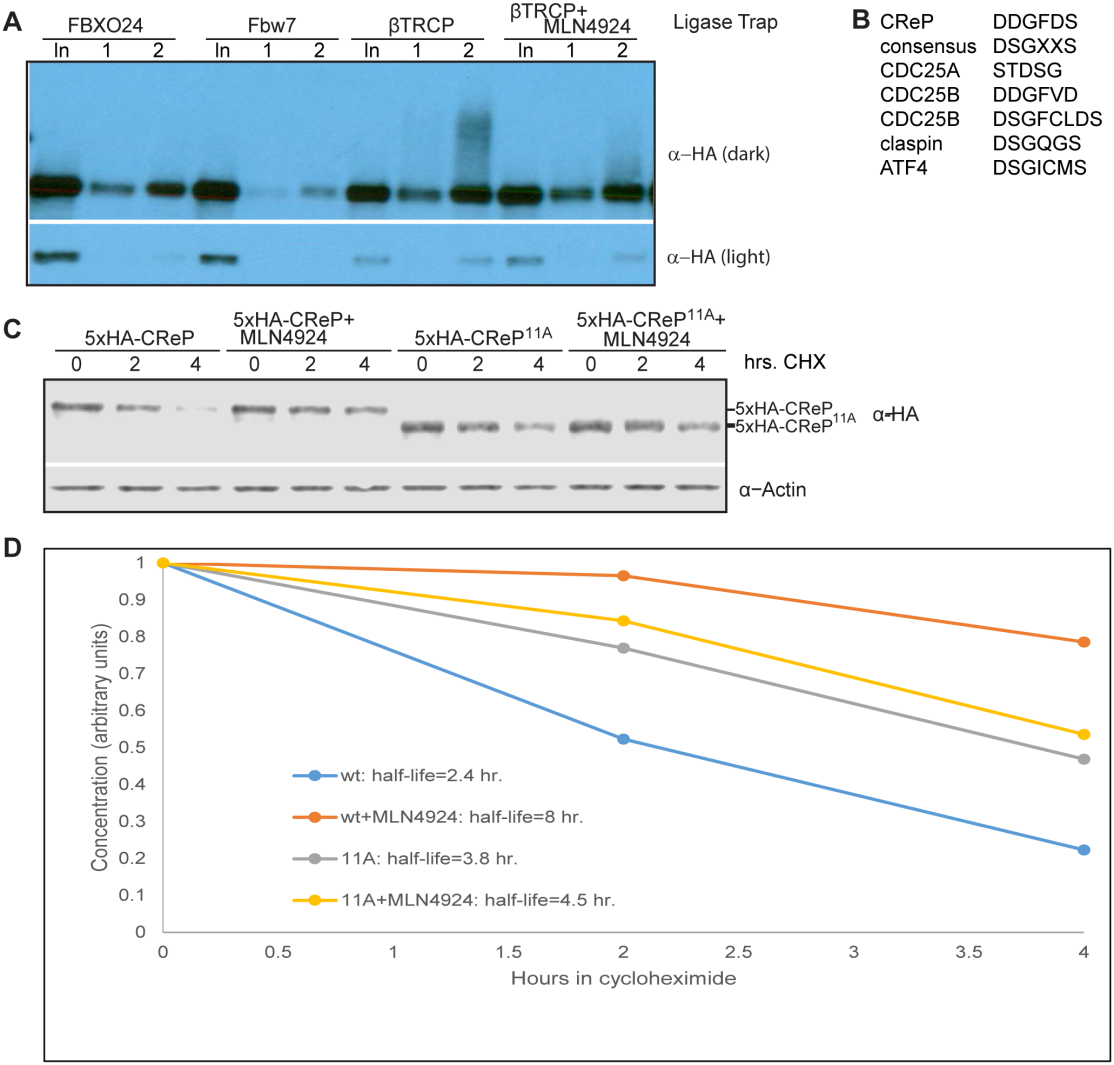
CReP ubiquitination is dependent on CRLs and turnover is regulated by a βTRCP consensus degron. (A) Ubiquitinated CReP precipitated by the βTRCP ligase trap depends on cullin activity. Tagged CReP was transiently expressed in the βTRCP or negative control ligase trap cell lines, as in Fig 2B. Where indicated, 1 μM MLN4924 was added 4 hours before cell collection to inhibit cullin activity. (B) A near-consensus βTRCP degron in CReP, compared to well-validated degrons. (C) CReP turnover depends on βTRCP consensus sites. Two consensus sites in CReP were mutated to generate the 11A mutant. Wildtype or mutant CReP was expressed transiently in 293 cells, which were then treated with 100 μg/mL cycloheximide for the time indicated to monitor degradation in the absence of new protein synthesis. Where indicated, cells were treated with 1 μM MLN4924 coincident with cycloheximide addition. (D) Quantitation of the average of two independent replicates of (B).

We wished to further explore the biological significance of CReP turnover. First, we verified that the ubiquitinated CReP pulled down by the βTRCP ligase trap required SCF activity. Indeed, pre-treatment of cells with MLN4924 eliminated the ubiquitinated CReP (but not unmodified CReP) pulled down by the βTRCP ligase trap (Fig 4A). Second, we mutated CReP’s single well-conserved βTRCP-binding consensus, as well as the amino acids immediately downstream, which form a second less-well-conserved consensus. The βTRCP consensus is DpSGX(1–4)pS [46], with some substitution of acidic amino acids for phosphorylations tolerated. The sequence we mutated in CReP is DDGFDSDSSLSDSD (marked in S11 Fig). Although this sequence lacks the most-conserved DSG motif, many well-documented βTRCP substrates have variations in this part of the degron [18], and human CDC25A and CDC25B have well-validated degrons that contain DDG, just like CReP [25] (shown in Fig 4B). This mutant, CReP^11A^, was significantly stabilized relative to wild type CReP (Fig 4C and 4D), strongly suggesting that CReP turnover is dependent on βTRCP. The notable downshift of the mutant is likely due to mutation of several negatively-charged residues. Mutation of a portion of the same region was independently shown to stabilize CReP while our manuscript was in the review process[47].

**Fig 5:**
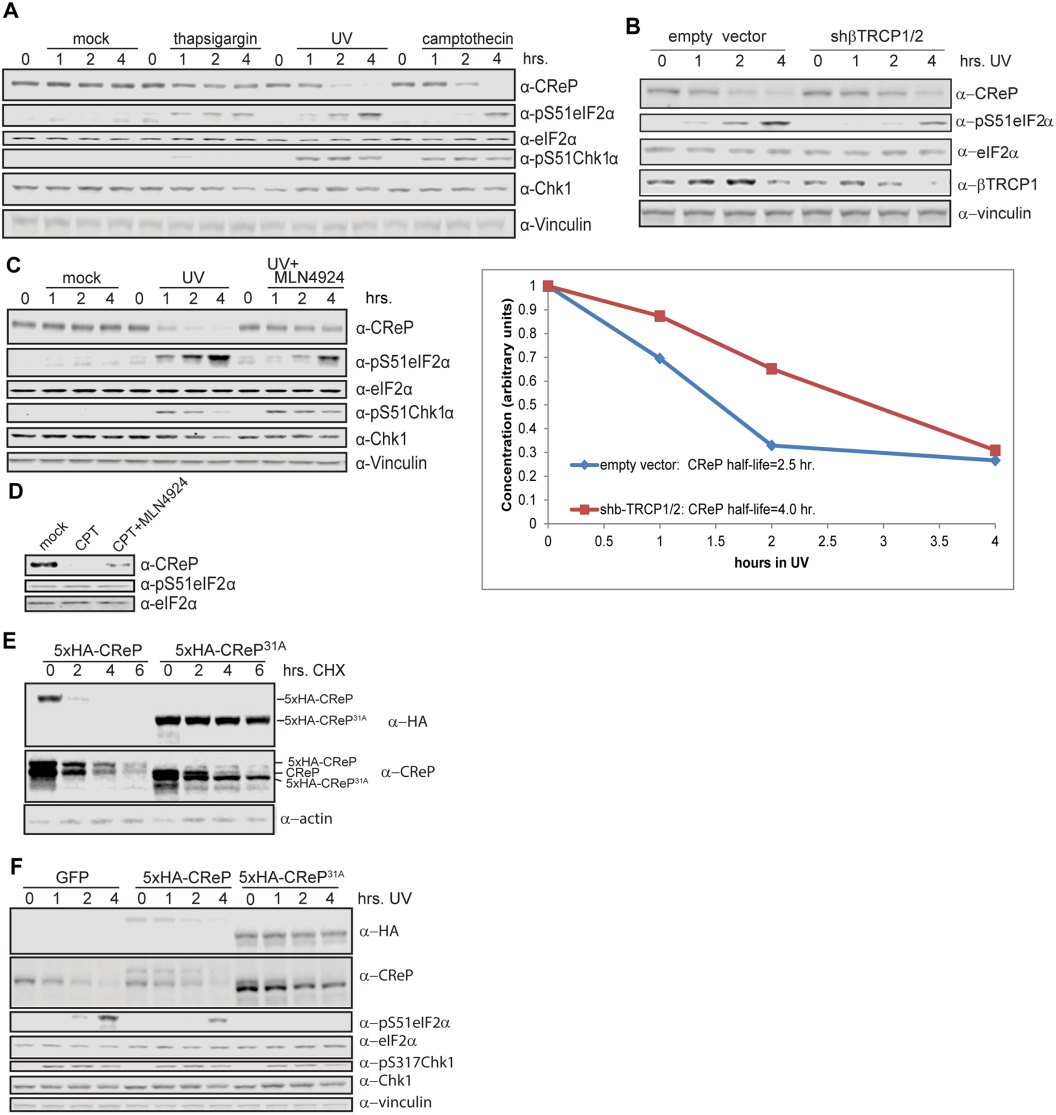
Regulation of CReP turnover and impact on eIF2α phosphorylation. (A) CReP is depleted upon DNA damage but not proteostatic stress. Cells were treated with 1 μM thapsigargin, 3 μg/mL camptothecin, or 300 J/m^2^ UV for the indicated time; all samples not treated with UV were mock-treated and all samples were given the same total volume of the solvent DMSO. (B) CReP turnover upon DNA damage depends at least in part on βTRCP. Cells were transfected for 48 hours with an empty vector or shRNA targeting βTRCP1 and 2, then irradiated with 300 J/m^2^ UV-C. CReP levels are quantitated below, and the half-life calculated from the linear (0–2 hr.) part of the timecourse. (C) CReP depletion and full eIF2α phosphorylation in UV depends on CRLs. Cells were treated with UV with or without MLN4924 for the times indicated. (D) CReP depletion in primary human fibroblasts depends on CRLs. Primary human fibroblasts were treated with 1 μg/mL camptothecin for 6 hours, with 1 μM MLN4924 where indicated. (E) The 31A allele of CReP is stable even upon treatment with DNA damage and cycloheximide. Cells were transfected with wildtype or mutant CReP, then pre-treated for 2 hours with 3 μg/mL camptothecin before addition of cycloheximide. (F) Expression of a stable allele of CReP prevents phosphorylation of eIF2α in response to UV treatment. Cells were transfected with tagged wild type or mutant CReP, then treated with UV for the indicated times.

Because both protein-folding stress and DNA damage have been shown to regulate eIF2α phosphorylation, we tested whether these stresses also regulated CReP levels. The proteostatic stress inducer thapsigargin had a very minor effect on CReP levels, consistent with a previous report showing no effect [34]. However, DNA damage provoked by either ultraviolet light (UV) or the topoisomerase inhibitor camptothecin (CPT) led to complete depletion of CReP (Fig 5A). Suggestively, the disappearance of CReP was coincident with the induction of eIF2α phosphorylation by these stressors. The depletion of CReP was not due merely to inhibition of translation by eIF2α phosphorylation, as DNA damage also decreases the half-life of CReP compared to no treatment or treatment with proteostatic stressors in a cycloheximide chase (S11 Fig), and CReP still disappears upon DNA damage in mouse embryonic fibroblasts in which Ser51 of eIF2α has been mutated to alanine (data not shown). CReP turnover and subsequent eIF2α phosphorylation is at least partially dependent on βTRCP, as transfection with shRNA against both paralogs of this ligase delays DNA damage-dependent induction of both CReP turnover and eIF2α phosphorylation (Fig 5B). CReP depletion is fully dependent on CRL-mediated degradation, because treatment of cells with the CRL inhibitor MLN4924 prevents CReP depletion (Fig 5C). The residual CReP turnover seen even in cells treated with βTRCP shRNA may reflect our inability to achieve sufficient knockdown of βTRCP, or additional turnover mediated by another CRL. Cullin-mediated turnover of CReP in response to DNA damage was not restricted to HEK293 cells, since it occurs in both primary human fibroblasts (Fig 5D) and immortalized mouse embryonic fibroblasts (MEFs) (S12 Fig).

The CReP^11A^ mutant was not completely stabilized upon DNA damage (data not shown), possibly because DNA damage promotes βTRCP binding to additional sites on CReP. βTRCP has been shown to interact with non-consensus phosphodegrons in MDM2, suggesting that it may be difficult to identify degrons by sequence alone[48]. Therefore, we mapped phosphorylated residues on CReP to identify any additional degron sequences (S9 Fig). Notably, most phosphosites were observed both with and without CPT. It is possible that the increase in CReP turnover observed upon DNA damage is not due to increased phosphorylation, but to a change in a targeting factor or localization of CReP. However, phosphosites are still likely to be required for turnover. For clustered phosphosites and phosphosites that were near short acidic stretches, we mutated both the phospho-acceptor and all acidic and potential phospho-acceptors in the region. In addition, we mutated one additional weak βTRCP consensus site that was not covered in the phospho-mapping. We then tested the stability of these mutants, in various combinations, in DNA damage (data not shown). CReP^31A^ (S10 Fig) was the least mutated allele that was completely stable upon treatment with DNA damage (Fig 5E and 5F). Importantly, this stabilization was not merely an artifact of high starting levels resulting from prioritized transcription or translation, as CReP^31A^ is stable even upon pre-treatment with camptothecin followed by cycloheximide chase (Fig 5E). Like the 11A mutant, CReP^31A^ migrates much more quickly than the endogenous protein, likely due to mutation of many negatively-charged amino acids.

**Fig 6:**
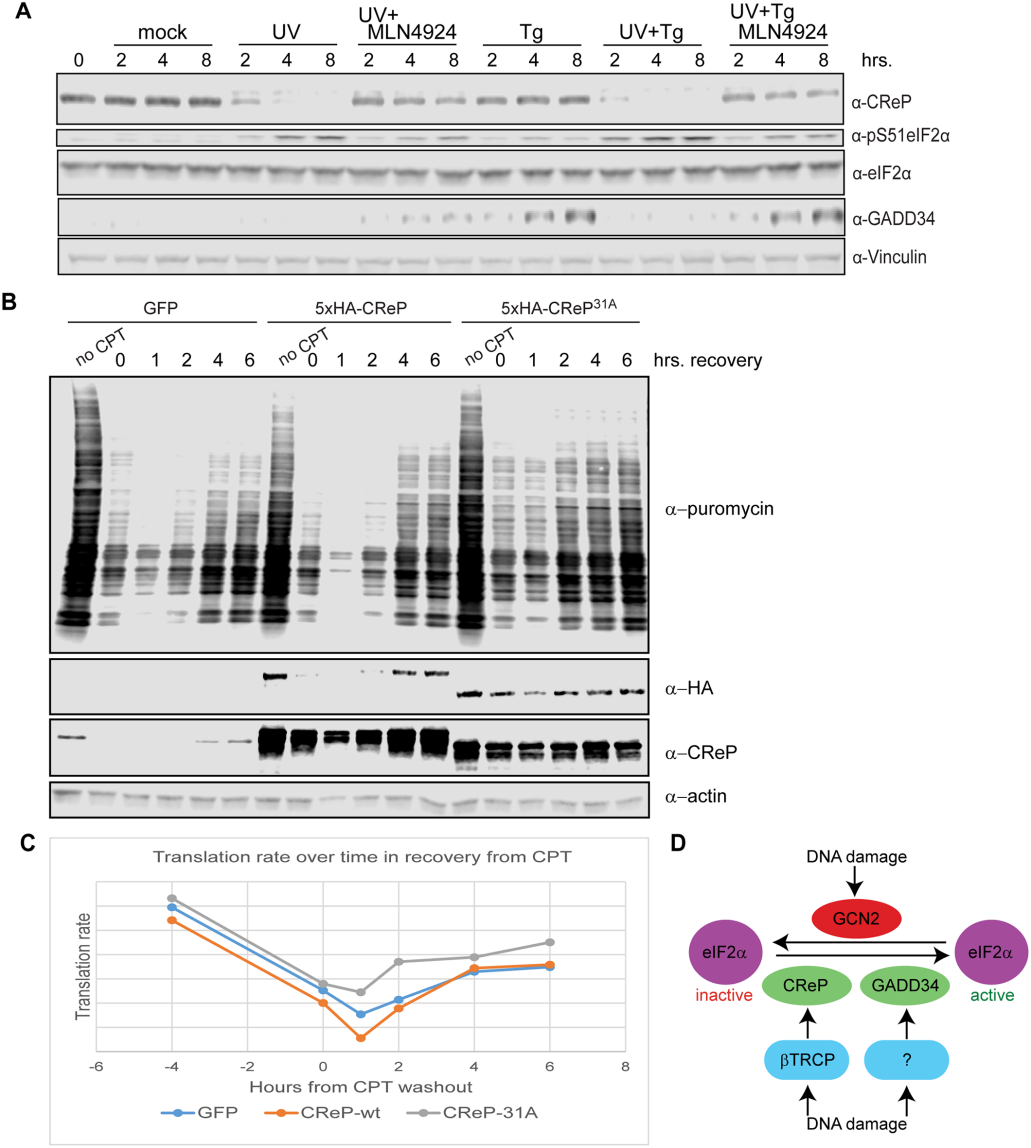
Consequences of CReP turnover downstream of eIF2α phosphorylation. (A) UV dominantly prevents the induction of GADD34. 293 cells were treated with 300 J/m^2^ UV-C, 1 μM thapsigargin, or a combination of the two, and with 1 μM MLN4924 where indicated. All treatments were added simultaneously. (B) CReP turnover reduces bulk translation after DNA damage. HEK293 cells were transfected with plasmids expressing GFP, wildtype CReP, or stable mutant CReP^31A^, then, as indicated, were untreated, treated with 1 μg/mL camptothecin (CPT) for 4 hours, or treated with CPT and then washed in medium to initiate DNA damage recovery for the indicated time. 10 minutes before collection, puromycin was added to cells at a final concentration of 10 μg/mL to label nascent polypeptide chains, and cells were collected in cold PBS, on ice, before flash-freezing. (C) Quantitation of (B) by densitometry. (D) A model for the role of CRLs in regulating eIF2α after DNA damage.

To examine the physiologic role of the turnover of CReP upon DNA damage, we determined whether CReP stabilization had an effect on eIF2α phosphorylation. When CReP turnover was inhibited by knockdown of βTRCP, treatment with MLN4924, or mutation of CReP, phosphorylation of eIF2α was delayed or inhibited to an equivalent degree (Fig 5B, 5C and 5F). This is not specific to HEK293 cells, as MLN4924 also reduced eIF2α phosphorylation after UV treatment in immortalized mouse embryonic fibroblasts (MEFs) (S12 Fig). However, primary human fibroblasts (Fig 6D) had constitutively high levels of eIF2α phosphorylation, so the effect of CReP turnover was only subtle. This may reflect a greater need for this pathway in fast-growing cells, or the fact that these primary cells were under constant stress.

Upon proteostatic stress, eIF2α phosphorylation promotes the translation of the transcription factor ATF4 [49]. ATF4 activates the expression of the transcription factor CHOP [49], which in turn promotes the transcription of GADD34 [50]. Like CReP, GADD34 is a PP1 targeting subunit that acts on Ser51 of eIF2α [51,52]. These PP1 subunits appear to have a dedicated role in regulating eIF2α, since the lethal phenotype of knockout mice lacking both GADD34 and CReP can be rescued by mutating eIF2α Ser51 [51]. Previous reports suggested that GADD34 is induced at late time points after DNA damage in some cell types [53]. We were especially interested in whether DNA damage promoted the destruction of CReP only to replace it with GADD34. However, we found that UV treatment did not promote GADD34 protein expression, while ER stress induced by thapsigargin did (Fig 6A). This may reflect a cell-type difference between HEK293 cells and cells previously used to show GADD34 induction. Surprisingly, treating cells with UV and thapsigargin simultaneously blocked the thapsigargin-mediated increase in GADD34 protein levels, suggesting that DNA damage somehow dominantly prevents expression of this protein. Inhibition of GADD34 expression by UV treatment could be rescued by simultaneously treating cells with MLN4924, suggesting that a CRL is involved in blocking GADD34 accumulation.

Finally, we examined whether CReP turnover after DNA damage affected rates of translation. After treatment with DNA damage, translation rate was assayed via the SUnSET method [54], by adding puromycin to the cells for 10 minutes, then detecting the degree of puromycin incorporation into newly translating polypeptides via western blotting with an anti-puromycin antibody. We found that expression of CReP^31A^, which led to high CReP levels even after treatment with camptothecin and initial recovery from this damage, accelerated the recovery of translation after DNA damage, doubling the translation rate at 2 hours after CPT washout (Fig 6B and 6C). Notably, this effect was not seen with the unstable, ectopically expressed wildtype CReP, although it was expressed at the same level as CReP^31A^. This effect reproduced several times, although the exact timing varies, likely due to subtle variations in CReP expression levels during transfection.

## Discussion

We have identified and validated thirteen novel substrates of the well-studied ubiquitin ligase βTRCP via Ubiquitin Ligase Trapping. While we were unable to test two of the twenty-eight candidate substrates identified, 88% of the remaining twenty-six were either known or validated novel substrates. While affinity chromatography is often able to identify ligase substrates, these data suggest that Ligase Trapping provides an unprecedented hit rate, making it an especially efficient way to identify new ubiquitin ligase substrates. Moreover, this technology has allowed us to easily validate substrates even if their bulk stability is not affected by βTRCP ubiquitination.

Our results for FBW7 suggest another way in which Ligase Trapping can complement currently available techniques. In a previous study, the Clurman lab pulled out all 26 members of the Mediator complex with FBW7. They used degron prediction and follow-up experiments to identify MED13 and MED13L as the ubiquitylated Fbw7 substrates and carefully confirmed that they are direct substrates. Our mass spec of the Fbw7 ligase trap immunoprecipitation specifically purified MED13 (and MED13L) uniquely in the Fbw7 Ligase Trap, and not in any of the other purifications. Moreover, we pulled out none of the other 25 subunits. This underscores the usefulness of our technique, especially for the great majority of F box proteins for which no degron consensus is known. Thus, even in cases where Ligase Trapping identifies similar numbers of substrates compared to other techniques, it allows one to quickly identify the directly ubiquitylated substrates.

In addition to the substrate CReP, which we followed up in detail, turnover of several of the other substrates is likely to be regulated in response to cell cycle position or stress. Sun2 is a transmembrane protein that spans the inner nuclear envelope and has been implicated in the maintenance of nuclear structure and the regulation of DNA damage. Its turnover by βTRCP may regulate these processes, and its removal from the membrane after ubiquitination may also be a regulated step. Strikingly, four of the eleven novel substrates we validated, ZNF395, HIVEP1/2, ZNF704, and AEBP2, are transcription factors, as are several known βTRCP substrates, such as Nrf2 and ATF4. We also identified two substrates that are themselves ubiquitin ligases, UBE4B and TRIM9, which opens up the possibility of complex mutual regulation. While UBE4B ubiquitination depends on the SCF (data not shown), it is not highly ubiquitinated (Fig 2), and it appears that the majority of the UBE4B population is stable (Fig 3B). RASSF3 is a candidate tumor suppressor protein that activates p53-dependent apoptosis under appropriate conditions, including DNA damage [55]. Its regulation by βTRCP is consistent with the known role of βTRCP in responding to DNA damage, and may help explain the oncogenic effect of βTRCP overexpression [18] (along with other known tumor suppressor substrates of βTRCP, such as REST[45]). RASSF3 appears to have both stable and unstable pools. This may reflect the relatively small pool of cells undergoing stress at any particular time in an untreated culture. Perturbations such as DNA damage might drive RASSF3 turnover.

Our previous studies in yeast [13] showed that 56% of newly-identified SCF substrates were strongly stabilized when the F box in question was mutated. 25% showed small or moderate stabilization, but were still unstable in the F box gene mutant. Finally, 19% appeared stable even in wildtype. We find here that 45% of confirmed novel substrates were stabilized by treatment with a pan-CRL inhibitor, 18% showed no stabilization, and 27% were stable in wildtype. Thus, in both cases only half or fewer novel substrates were quantitatively turned over by the single ligase, although this is likely an underestimate overall, since previously characterized substrates may be biased for this category. While some of these effects could be due to the population assay employed, as noted above, substrates such as Cln3 and Gal4 in yeast, as well as PIP box-containing substrates in humans, are targeted in a way that is dependent upon the sub-cellular localization/context of the substrate [12,56]. Alternatively, some turnover events occur as part of quality control pathways that only target those proteins that are in some way defective.

We have implicated βTRCP in the regulation of translation initiation after DNA damage through its turnover of CReP, and shown that DNA damage-induced phosphorylation of eIF2α, because it uniquely requires the depletion of CReP, occurs via a different mechanism from the other stresses known to promote eIF2α phosphorylation, which all promote kinase activation. Previous work has shown that the phosphorylation of eIF2α after UV treatment depends on the kinase Gcn2 [57,58]. We propose that this phosphorylation requires both Gcn2 activation and CReP turnover.

Why does phosphorylation of eIF2α require CReP depletion after DNA damage, but not in response to proteostatic stress? One possibility is that eIF2alpha kinases are less active after DNA damage than after proteostatic stress. We observed that, once CReP levels begin to drop, eIF2α phosphorylation is much higher upon our UV treatment than after proteostatic stress (Fig 5A). This likely reflects both continued CReP activity and the induction of GADD34 upon proteostatic stress. We showed in Fig 6B and 6C that CReP turnover has a significant effect on translation rates after DNA damage, but substantial inhibition of translation happens even in the absence of CReP turnover. Translation rates are highly redundantly regulated, both via control of eIF2α phosphorylation and via regulation of eIF4. Our results are consistent with a model in which CReP turnover is important to enforce continued low levels of translation at later timepoints. Moreover, the high levels of eIF2α phosphorylation enabled by CReP turnover in response to DNA damage may allow translational reprograming that leads to induction of DNA damage repair proteins, even as global translation is downregulated. Indeed, translation of several DNA repair proteins has been shown to be resistant to inhibition of CAP-dependent translational inhibition by eIF2α phosphorylation [58].

Finally, how do CRLs prevent the induction of GADD34 after UV treatment? One possibility is that CReP turnover upon DNA damage (which requires CRLs) drives such strong eIF2α phosphorylation that translation of GADD34 or one of its upstream regulators ATF4 or CHOP is inhibited. Another possibility is that a CRL is turning over a specific protein to keep GADD34 levels low. βTRCP is known to target ATF4 [24] and the Cul3-associated ligase SPOP is reported to target CHOP [59]. GADD34 is also a known proteasome target, consistent with its being a substrate of βTRCP or another CRL [60]. Targeting of both CReP and Gadd34 for degradation upon DNA damage underscores the importance of limiting eIF2α phosphatase activity during DNA damage.

## Methods

### Plasmids and tissue culture

All plasmids were transfected into the 293 FlpIn TRex cell line (Life Technologies, Grand Island, NY, USA), which contains both a site for FRT-mediated recombination (which we did not use in this work) and expresses the *tet* repressor, which allows doxycycline-inducible expression from promoters that include *tet* operators. Mouse embryonic fibroblasts (MEFs) were immortalized by transduction with the SV40 large T antigen (kind gift of Morgan Truitt and Davide Ruggero). All cells were grown in DMEM with 10% heat-inactivated fetal bovine serum. For large-scale purifications, medium was supplemented with 500 U/mL penicillin and 500 μg/mL streptomycin.

6xHis-ubiquitin was expressed from pTB30, a modified pcDNA3.1 vector with a pCMV/TetO promoter expressing 6xHis-Uba52-IRES-6xHis-RPS27A. The parent of this construct was the kind gift of Zhijian Chen, UT Southwestern. The construct was linearized with *Pvu I* and transfected into 293 FlpIn TRex cells. Stable transfectants were selected with G418 and a clone was selected that expressed at a high level only upon treatment with doxycycline.

To make the ligase trap fusion proteins, F box proteins were fused on the C-terminus to 3xFlag followed by the C terminal half of human RAD23B (Accession #BC020973.2, amino acids 185–410), encoding two UBA domains. Ligase traps βTRCP2 (FBXW11; Accession #BC026213.1, pTB53), Fbxo24 (Accession #NM033506.2, pBEN20), and Fbxo6 (Accession #NM018438.5, pBEN5) were expressed as hygromycin resistance-T2A-ligase trap fusions driven by the mouse PGK1 promoter. Each of these constructs also expresses an shRNA against the relevant F box protein (to which the fusion protein is resistant), driven by the mouse U6 promoter. These cassettes were linearized by digestion with *Pac I*. Fbw7 (Accession# NM_033632.3, pTB59) Ligase Trap was expressed from a pcDNA3.1 vector, under the control of the CMV promoter. The vector was linearized with *BglII*. All linearized plasmids were transfected into the HisUb cell line and stable transfectants were selected with hygromycin. We selected clonal cell lines that expressed moderate levels of the relevant ligase trap.

All substrate proteins were tagged on the N-terminus with the 5xHA epitope, and expressed from the CMV promoter in pcDNA3.1, except SUN2, AEBP2, ALDH2, and RASSF3, which were tagged on the C-terminus. They were transiently transfected into the relevant cell line using Fugene HD at 3 μL/μg DNA (Promega Corporation, Madison, WI, USA) or polyethyleneimine (at 18 μg/μg DNA) 24–48 hours before the experiment. βTRCP was knocked down with an shRNA targeting both BTRC and FBXW11, expressed from the pSUPER-puro-retro vector (under the H1 promoter)[61].

### Drugs

MG132 is used at 5 μM. MLN4924 is used at 1 μM. Camptothecin is used at 3 μg/mL, unless otherwise specified.

### UV treatment

Medium was removed from adherent cells and set aside. Cells were covered in 37°C 1X PBS with 0.9 mM CaCl_2_ and 0.5 mM MgCl_2_, then exposed to 300 J/m^2^ UV-C, PBS was aspirated, and medium was replaced.

### Antibodies and western blotting

For western blotting, cells were lysed in 1X RIPA buffer with protease and phosphatase inhibitors for 30 minutes on ice, insoluble material was spun out, then protein concentrations were measured with BCA Reagent (Pierce, Thermo Scientific, Rockford, IL, USA) and normalized before addition of SDS sample buffer with DTT. For Figs S7 (except for RASSF3) and 5C, cells were lysed directly in SDS sample buffer with DTT or βMe.

All gels were Criterion Tris-HCl 4–20% gradients (cat. #345–0034, BioRad, Hercules, CA, USA), except for the gel for the α-HA blot in Fig 2C, which was a 7.5% gel (BioRad cat. #345–0007).

Antibodies used were α-HA 16B12 at 1:1,000–1:2,000 (cat. #MMS-101R, Covance, Emeryville, CA, USA), α-6xHis at 1:1,000–1:2,000, α-ubiquitin P4D1 at 1:100, α-Flag M2 at 1:2,000 (cat. #F3165, Sigma, St. Louis, MO, USA), α-Cul1 at 1:1,000, α-vinculin at 1:1,000–1:5,000, α-βactin at 1:1,000–1:10,000 (Sigma cat. #A5441 for Fig 4A, Abcam, Cambridge, UK, cat.#ab8226 for all others), α-PPP1R15B (CReP) at 1:1,000–1:5,000 (cat. #14634-1-AP, Proteintech, Chicago, IL, USA), and α-GADD34 (cat. # 10449-1-AP, Proteintech, Chicago, IL, USA). α-phosphoS51-eIF2α (cat. #9721), α-eIF2α (cat. #9722), α-phosphoS317Chk1 (cat. #2344), and α-Chk1 (cat. #2360) antibodies were all from Cell Signaling Technologies, Danvers, MA, USA. The α-puromycin antibody 12D10 was from EMD Millipore (cat. #MABE343).

Western blots in Figs 1, 2A, 2B and 3A were incubated with secondary antibodies fused to horseradish peroxidase and visualized by treatment with Western Lightning ECL (Perkin Elmer, Waltham, MA, USA). Western blots in Figs 2C, 3B and 4 were incubated with fluorescent secondary antibodies and visualized with an Odyssey scanner (Licor, Lincoln, NE, USA).

### Immunoprecipitations of Ligase Traps

Unless otherwise noted, stable cell lines expressing Ligase Traps were treated with 5 μM MG132 for 4 hours before collection. We grew 100–200 barely sub-confluent 15 cm dishes for each purification, representing approximately 1–3 x 10^9^ cells. Pellets were lysed in 25 mM Hepes-KOH, pH8, 150 mM K Oac,10 mM MgCl2, 5 mM CaCl2, 20 mM iodoacetamide, 30 μM MG132, protease inhibitors, and phosphatase inhibitors by sonication, then treated with DNase (660 U/mL) at 4°C for 30 minutes before addition of Nonidet P-40 to 0.1%. Samples were spun to remove insoluble material, then incubated with α-Flag M2 magnetic beads (Sigma, St. Louis, MO, USA) at 4°C overnight. Beads were washed 5 times in 1X PBS+0.1% Nonidet P-40, then eluted in this wash buffer+0.5 mg/mL 3xFlag peptide. The eluate was denatured by addition of 2X volume Buffer B (216 mM NaH_2_PO_4_, 16 mM Tris, 9.37 M urea, pHed to 8). The sample was then incubated with NiNTA agarose for 3 hours at room temperature. The beads were washed 3X in Buffer B diluted to 8M urea+10 mM imidazole, then 2X in Buffer B diluted to 1 M urea+10mM imidazole. Samples were eluted in 0.5 M urea, 300 mM imidazole, 0.1% rapigest (or Nonidet P-40 if not to be used for mass spectrometry), 108 mM NaH_2_PO_4_, 8 mM Tris (pHed to 8 before adding imidazole).

### Mass spectrometry analysis

The immunopurified protein complexes were mixed in a ratio of 1:1 with digestion buffer (100 mM Tris-HCl, pH 8.5, 8M urea), reduced, alkylated and digested by sequential addition of lys-C and trypsin proteases as previously described[62,63]. For identification of phosphorylation site, proteins were digested directly in the excised gel slice using trypsin[62]. Peptide digests desalted and fractionated online using a 50 μM inner diameter fritted fused silica capillary column with a 5 μM pulled electrospray tip and packed in-house with 15 cm of Luna C18(2) 3 μM reversed phase particles. The gradient was delivered by an easy-nLC 1000 ultra high pressure chromatography system (Thermo Scientific). MS/MS spectra were collected on a Q-Exactive mass spectrometer (Thermo Scientific) [64,65]. Data analysis was performed using the ProLuCID, DTASelect2, and Ascore algorithms as implemented in the Integrated Proteomics Pipeline—IP2 (Integrated Proteomics Applications, Inc., San Diego, CA) [66–69]. Phosphopeptides were identified using a differential modification search that considered a mass shift of +79.9663 on serines, threonines and tyrosines. Protein and peptide identifications were filtered using DTASelect and required at least two unique peptides per protein and a peptide-level false positive rate of less than 5% as estimated by a decoy database strategy[70]. Normalized spectral abundance factor (NSAF) values were calculated as described and multiplied by 10^5^ to improve readability [71].

### Puromycin incorporation assay

We followed the SUnSET protocol [54]. Puromycin was added to culture medium at a final concentration of 10 μg/mL, incubated for 10 minutes at 37°C and 8% CO_2_, then medium was replaced with ice-cold PBS with 5 mM EDTA, and cells were sprayed from the dish on ice, spun down at 4°C and flash-frozen. Samples were normalized by protein concentration, and puromycin incorporation was detected by western blotting with a monoclonal anti-puromycin antibody (12D10) and quantified by densitometry.

## Supporting Information

S1 Fig Development of the mammalian Ligase Trapping protocol. (A) We created the 293 HisUb cell line, which expresses high levels of 6xHis-tagged ubiquitin upon doxycycline treatment, in addition to endogenous ubiquitin. We added doxycycline for 3 days and the proteasome inhibitor MG132 for 4 hours, where noted. (B) To choose a UBA domain to include in our Ligase Trap constructs, we fused UBA domains from 3 different sources to βTRCP. Cells were induced to express 6xHisUb with doxycycline, the transiently transfected with equal amounts of Ligase Trap constructs including βTRCP-3xFlag fused to the tandem UBA domains of RAD23B or RAD23A, the single UBA domain of ubiquilin 2, or Flag alone, and the total 6xHisUb pulled down by each construct was assayed. Cells were treated with 5 μM MG132 for 4 hours before lysis. The F box fusions were purified under native conditions with anti-Flag antibody and eluted with Flag peptide. Then, the eluate was denatured in 6M urea and ubiquitinated proteins purified with NiNTA beads and eluted with imidazole. Loading was 1X for input, 23X for the 1^st^ step, and 195X for the 2^nd^ step. (C) To determine the best course of MG132 treatment, we induced 6xHisUb expression and treated the stable cell line expressing the βTRCP-3xFlag-RAD23B Ligase Trap construct with 5 μM MG132 for 0, 2, or 4 hours before lysis. Loading was 1X for input, 20X for the 1^st^ step, and for the 2^nd^ step, 936X for the α-ubiquitin blot and 312X for the α-Flag blot.
(TIFF)

S2 Fig Purification of ubiquitinated β-catenin by the βTRCP Ligase Trap. Stable cell lines expressing the βTRCP Ligase Trap or a negative control (FBXO6) were induced to express 6xHisUb for 3 days, transfected with 5xHA-tagged β-catenin for 24 hours, lysed and subjected to a two-step precipitation. First, the Ligase Traps were purified under native conditions with anti-Flag antibody and eluted with Flag peptide. Then, the eluate was denatured in 6M urea and ubiquitinated proteins purified with NiNTA beads and eluted with imidazole. Loading was 1X input (In), 160X 1st step (1), and 1950 2nd step (2) for the a-HA blot and 1X input, 20X 1st step, and 170X 2nd step for the a-Flag and a-Cul1 blots.
(TIF)

S3 Fig Conservation of degrons observed in candidate βTRCP substrates. Comparison of degron sequences observed to the corresponding sequence in the mouse and chicken homolog.
(TIFF)

S4 Fig Pulldown of ubiquitinated species of candidate substrates by the βTRCP Ligase Trap. Complete IP results for candidate substrates shown in Fig 2, as well as for bHLHE40 and TFAP4, which are now listed as known substrates since they were published during the preparation of this manuscript.
(TIF)

S5 Fig Pulldown of ubiquitinated species of candidate substrates by the βTRCP Ligase Trap. As in Fig 2.
(TIF)

S6 Fig Determination of candidate substrate stability and effect of SCF inhibition. Effect of SCF inhibition on candidate substrate half-life. 293 cells were transiently transfected with 5xHA-tagged candidate substrates and then treated with 100 μg/mL cycloheximide (CHX) for the indicated time to halt protein synthesis. Where indicated, 1 μM MLN4924 was added at the same time as CHX. RASSF3 samples were all from the same blot and exposure.
(TIF)

S7 Fig Effect of MG132 on βTRCP Ligase Trap pulldowns. All substrates listed in Table 1 are included, with their average total spectral counts from three purifications in the presence of 5 μM MG132 and two purifications in the absence of MG132.
(TIF)

S8 Fig Accumulation of CReP upon CRL inhibition. HEK293 cells were treated with 1 μM MLN4924 for the indicated time, and CReP levels assayed.
(TIF)

S9 Fig Phospho-site mapping of CReP. 3xFlag-CReP was transiently expressed in 293 FlpInTRex cells, which were treated with 1 μM MLN4924 for 5 hours and, where noted, 3 μg/mL camptothecin for 4 hours before lysis. Then 3xFlag-CReP was purified with anti-Flag antibody, run on an SDS-PAGE gel, stained with colloidal Coomassie, and a band of the corresponding molecular weight was cut out. The gel slice was analyzed by mass spectrometry to identify phospho-sites. Predicted phospho-sites are shown for unstressed (A) and camptothecin-treated (B) cells. Coverage for unstressed (C) and camptothecin-treated (D) samples was about 40%.
(TIF)

S10 Fig Amino acid sequence of CReP, with stabilizing mutations marked. Residues mutated to alanine in the 11A mutant are marked in red. The 31A mutant includes those alanines as well as alanines in place of the residues marked in blue.
(TIF)

S11 Fig DNA damage decreases CReP half-life. Cells were treated with the indicated concentrations of the indicated drugs for 2.5 hours before addition of cycloheximide for the indicated time.
(TIF)

S12 Fig CRL activity is required for full CReP depletion and eIF2α phosphorylation after UV treatment in mouse embryonic fibroblasts (MEFs). Immortalized MEFs were treated with 300 J/m^2^ UV-C light for the indicated time, and simultaneously with 1 μM MLN4924 where indicated.
(TIF)

S1 Table Mass spectrometry data. All polypeptides identified in any of five βTRCP Ligase Trap purifications (three with and two without MG132) or any negative control purification. For each polypeptide found in any Ligase Trap purification, the total spectral counts, normalized to the total number of counts for that purification, is listed, along with the average number of background counts. The score listed for each condition is the average number of counts pulled down for that polypeptide in that condition divided by the average number of background counts.
(XLSX)

S2 Table Raw data underlying quantitations of western blots. In separate tabs, the raw data underlying Figs 4D, 5B and 6C.
(XLSX)
