# Supplementary figures and images for: DNA Damage Regulates Translation through β-TRCP Targeting of CReP
Source: PLoS Genet. 2015 Jun 19;11(6):e1005292. doi: 10.1371/journal.pgen.1005292 (PMC4474599; doi:10.1371/journal.pgen.1005292)

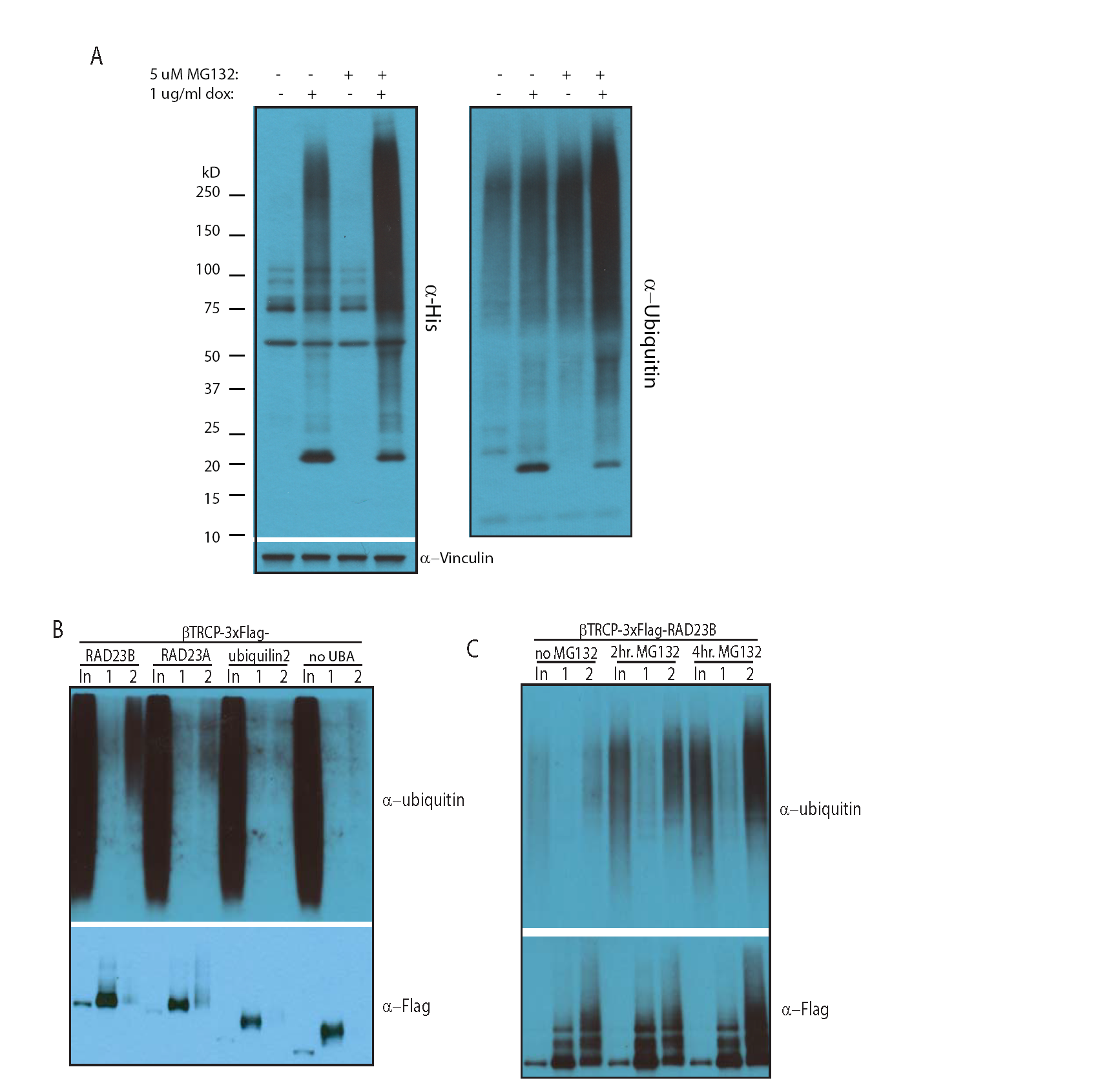

Supplement: S1 Fig — (A) We created the 293 HisUb cell line, which expresses high levels of 6xHis-tagged ubiquitin upon doxycycline treatment, in addition to endogenous ubiquitin. We added doxycycline for 3 days and the proteasome inhibitor MG132 for 4 hours, where noted. (B) To choose a UBA domain to include in our Ligase Trap constructs, we fused UBA domains from 3 different sources to βTRCP. Cells were induced to express 6xHisUb with doxycycline, the transiently transfected with equal amounts of Ligase Trap constructs including βTRCP-3xFlag fused to the tandem UBA domains of RAD23B or RAD23A, the single UBA domain of ubiquilin 2, or Flag alone, and the total 6xHisUb pulled down by each construct was assayed. Cells were treated with 5 μM MG132 for 4 hours before lysis. The F box fusions were purified under native conditions with anti-Flag antibody and eluted with Flag peptide. Then, the eluate was denatured in 6M urea and ubiquitinated proteins purified with NiNTA beads and eluted with imidazole. Loading was 1X for input, 23X for the 1st step, and 195X for the 2nd step. (C) To determine the best course of MG132 treatment, we induced 6xHisUb expression and treated the stable cell line expressing the βTRCP-3xFlag-RAD23B Ligase Trap construct with 5 μM MG132 for 0, 2, or 4 hours before lysis. Loading was 1X for input, 20X for the 1st step, and for the 2nd step, 936X for the α-ubiquitin blot and 312X for the α-Flag blot. (TIFF) [file pgen.1005292.s001.tiff]

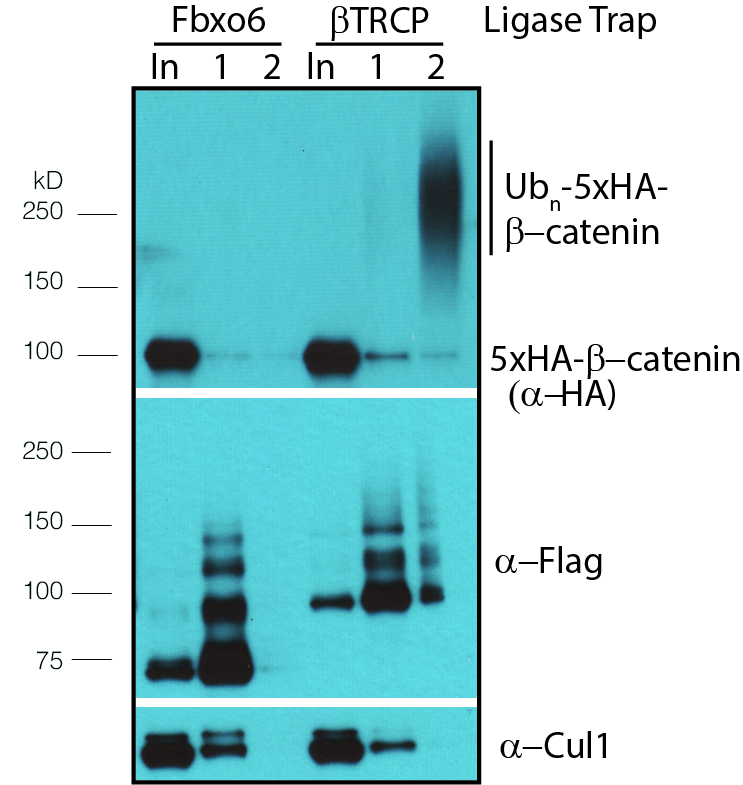

Supplement: S2 Fig — Stable cell lines expressing the βTRCP Ligase Trap or a negative control (FBXO6) were induced to express 6xHisUb for 3 days, transfected with 5xHA-tagged β-catenin for 24 hours, lysed and subjected to a two-step precipitation. First, the Ligase Traps were purified under native conditions with anti-Flag antibody and eluted with Flag peptide. Then, the eluate was denatured in 6M urea and ubiquitinated proteins purified with NiNTA beads and eluted with imidazole. Loading was 1X input (In), 160X 1st step (1), and 1950 2nd step (2) for the a-HA blot and 1X input, 20X 1st step, and 170X 2nd step for the a-Flag and a-Cul1 blots. (TIF) [file pgen.1005292.s002.tif]

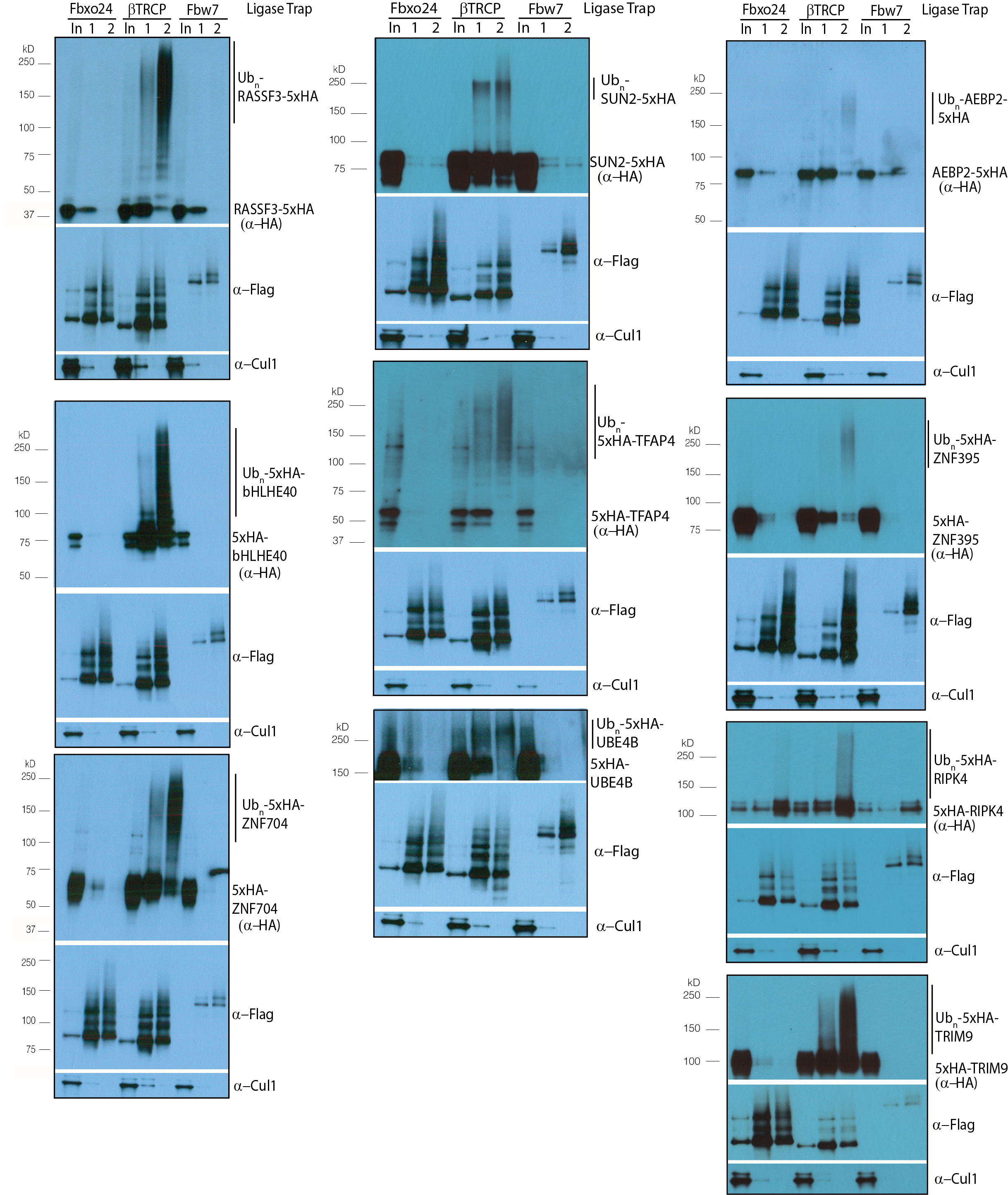

Supplement: S4 Fig — Complete IP results for candidate substrates shown in Fig 2, as well as for bHLHE40 and TFAP4, which are now listed as known substrates since they were published during the preparation of this manuscript. (TIF) [file pgen.1005292.s004.tif]

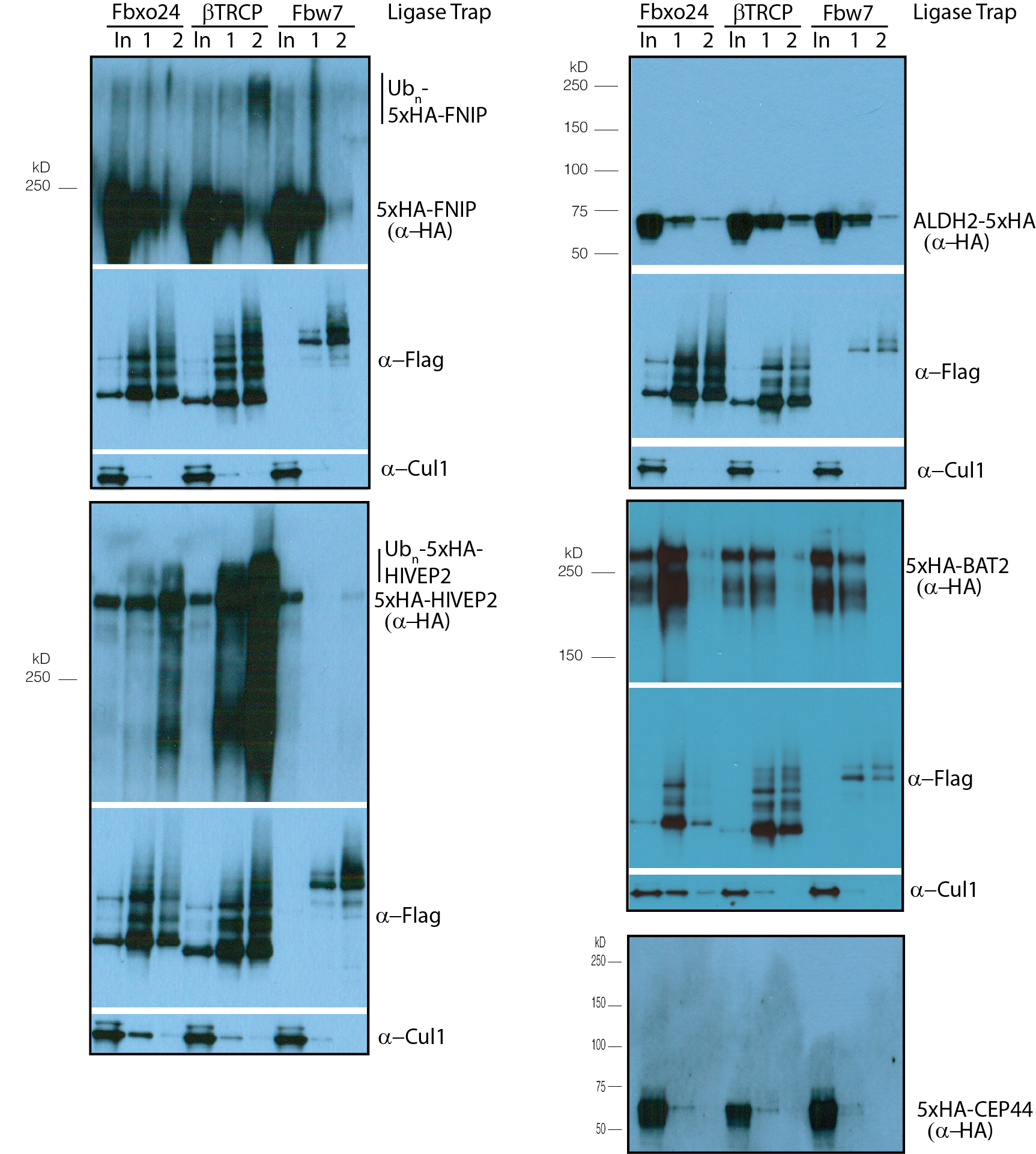

Supplement: S5 Fig — As in Fig 2. (TIF) [file pgen.1005292.s005.tif]

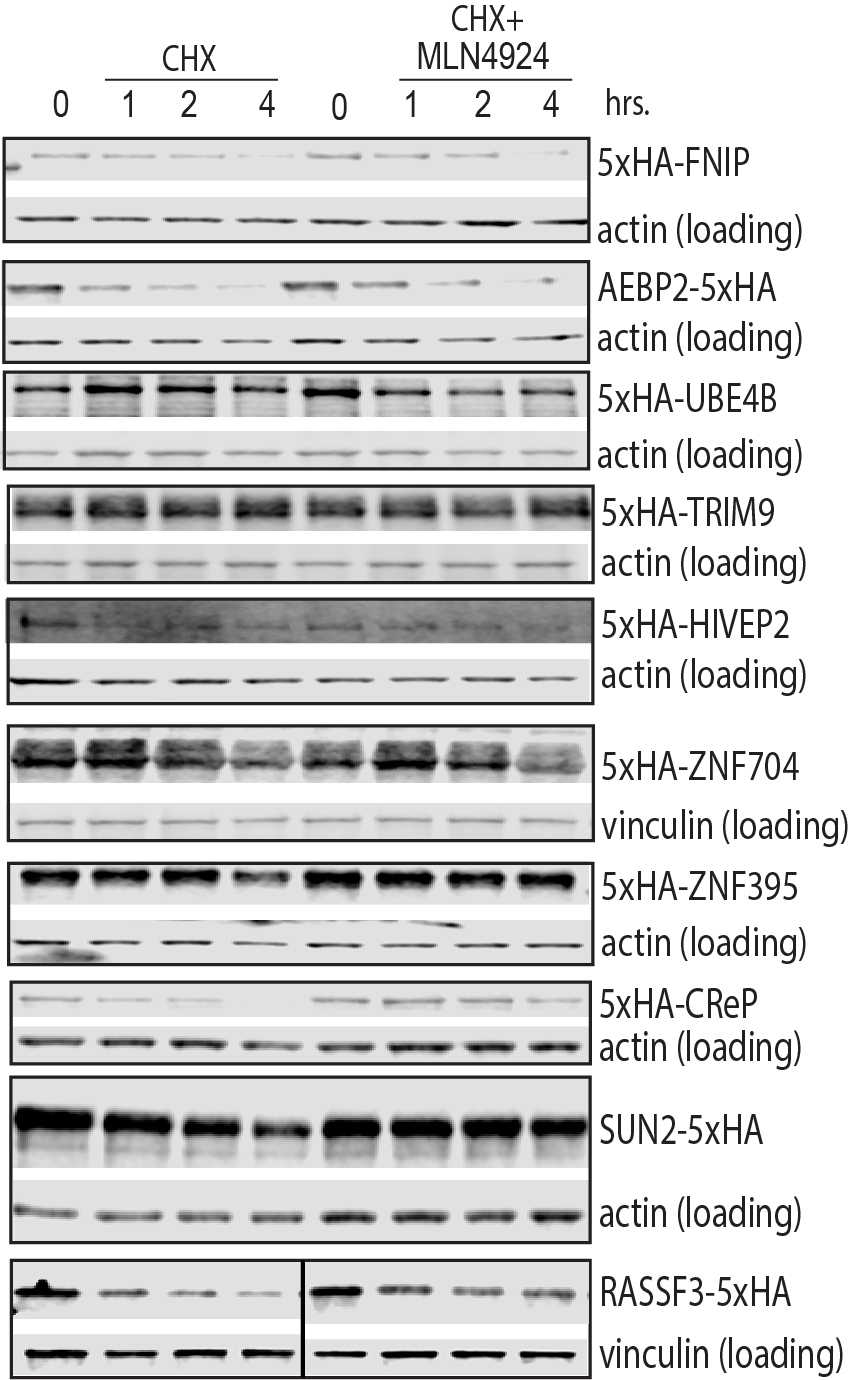

Supplement: S6 Fig — Effect of SCF inhibition on candidate substrate half-life. 293 cells were transiently transfected with 5xHA-tagged candidate substrates and then treated with 100 μg/mL cycloheximide (CHX) for the indicated time to halt protein synthesis. Where indicated, 1 μM MLN4924 was added at the same time as CHX. RASSF3 samples were all from the same blot and exposure. (TIF) [file pgen.1005292.s006.tif]

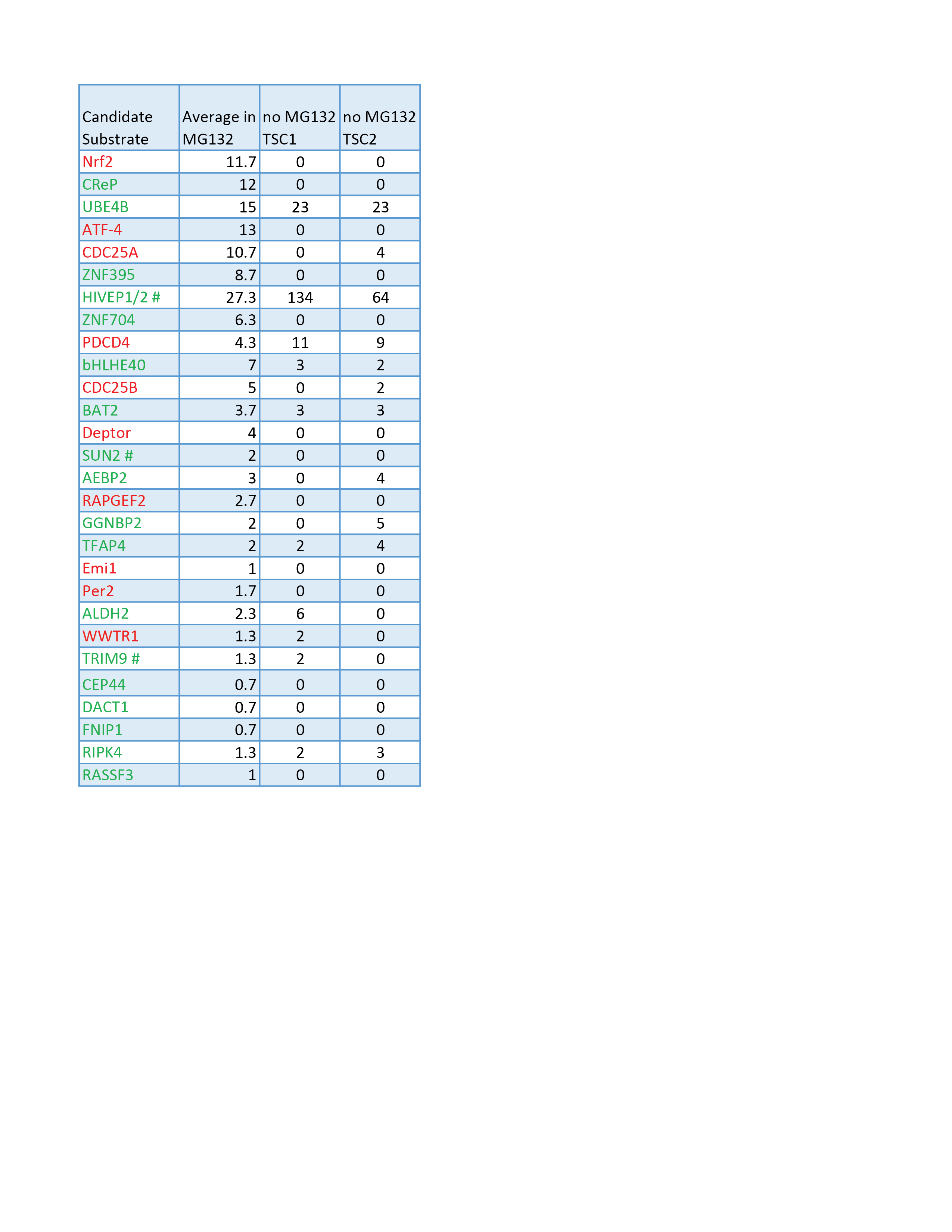

Supplement: S7 Fig — All substrates listed in Table 1 are included, with their average total spectral counts from three purifications in the presence of 5 μM MG132 and two purifications in the absence of MG132. (TIF) [file pgen.1005292.s007.tif]

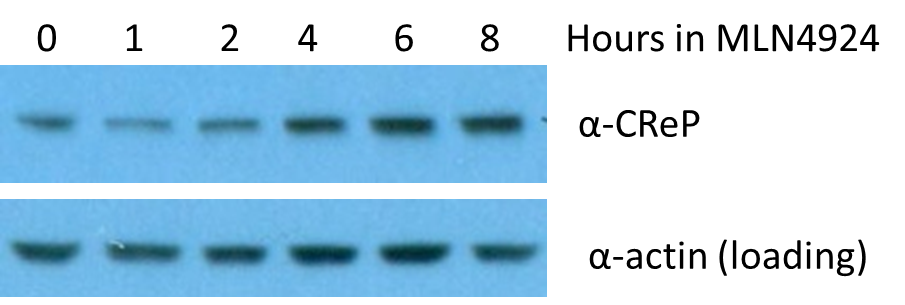

Supplement: S8 Fig — HEK293 cells were treated with 1 μM MLN4924 for the indicated time, and CReP levels assayed. (TIF) [file pgen.1005292.s008.tif]

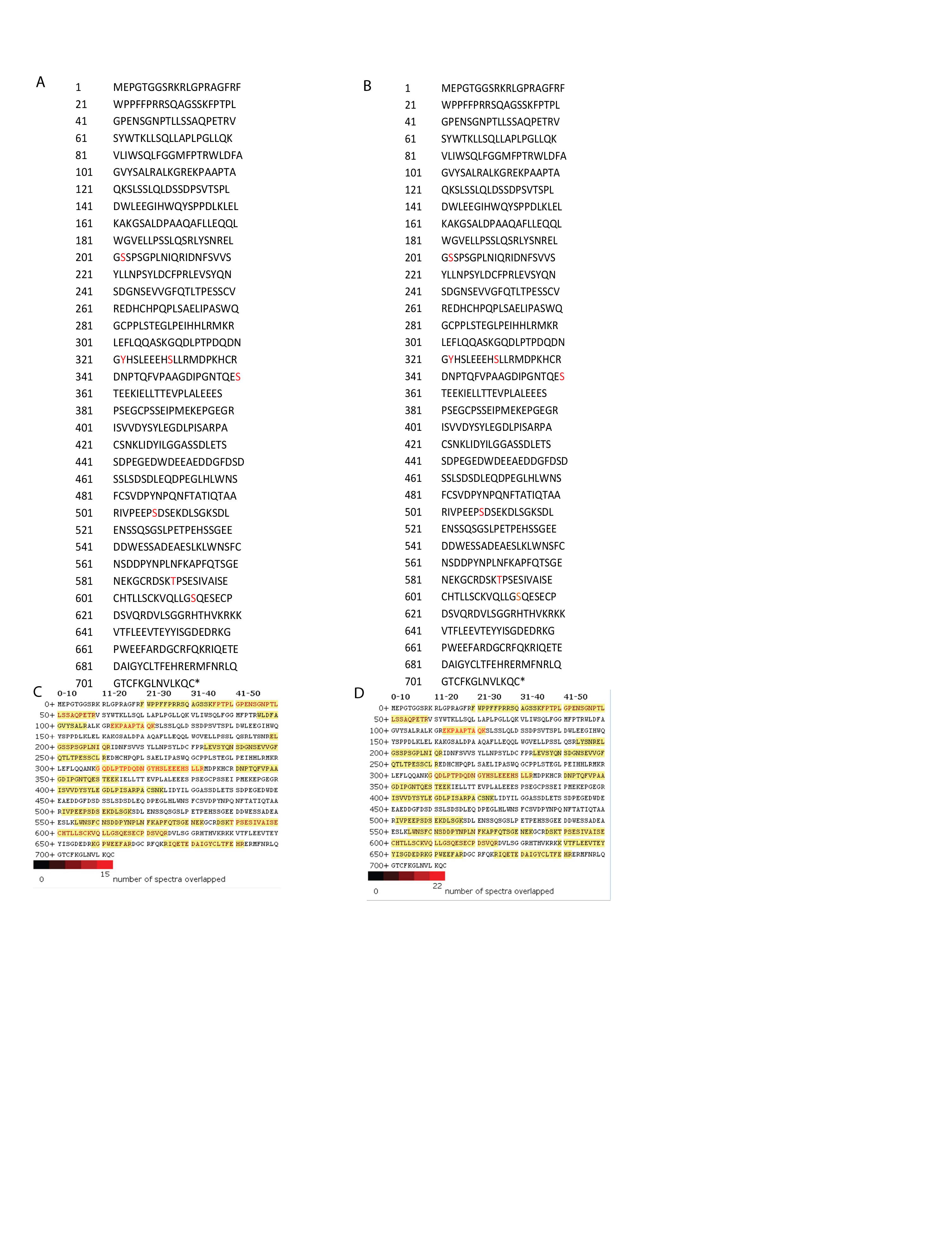

Supplement: S9 Fig — 3xFlag-CReP was transiently expressed in 293 FlpInTRex cells, which were treated with 1 μM MLN4924 for 5 hours and, where noted, 3 μg/mL camptothecin for 4 hours before lysis. Then 3xFlag-CReP was purified with anti-Flag antibody, run on an SDS-PAGE gel, stained with colloidal Coomassie, and a band of the corresponding molecular weight was cut out. The gel slice was analyzed by mass spectrometry to identify phospho-sites. Predicted phospho-sites are shown for unstressed (A) and camptothecin-treated (B) cells. Coverage for unstressed (C) and camptothecin-treated (D) samples was about 40%. (TIF) [file pgen.1005292.s009.tif]

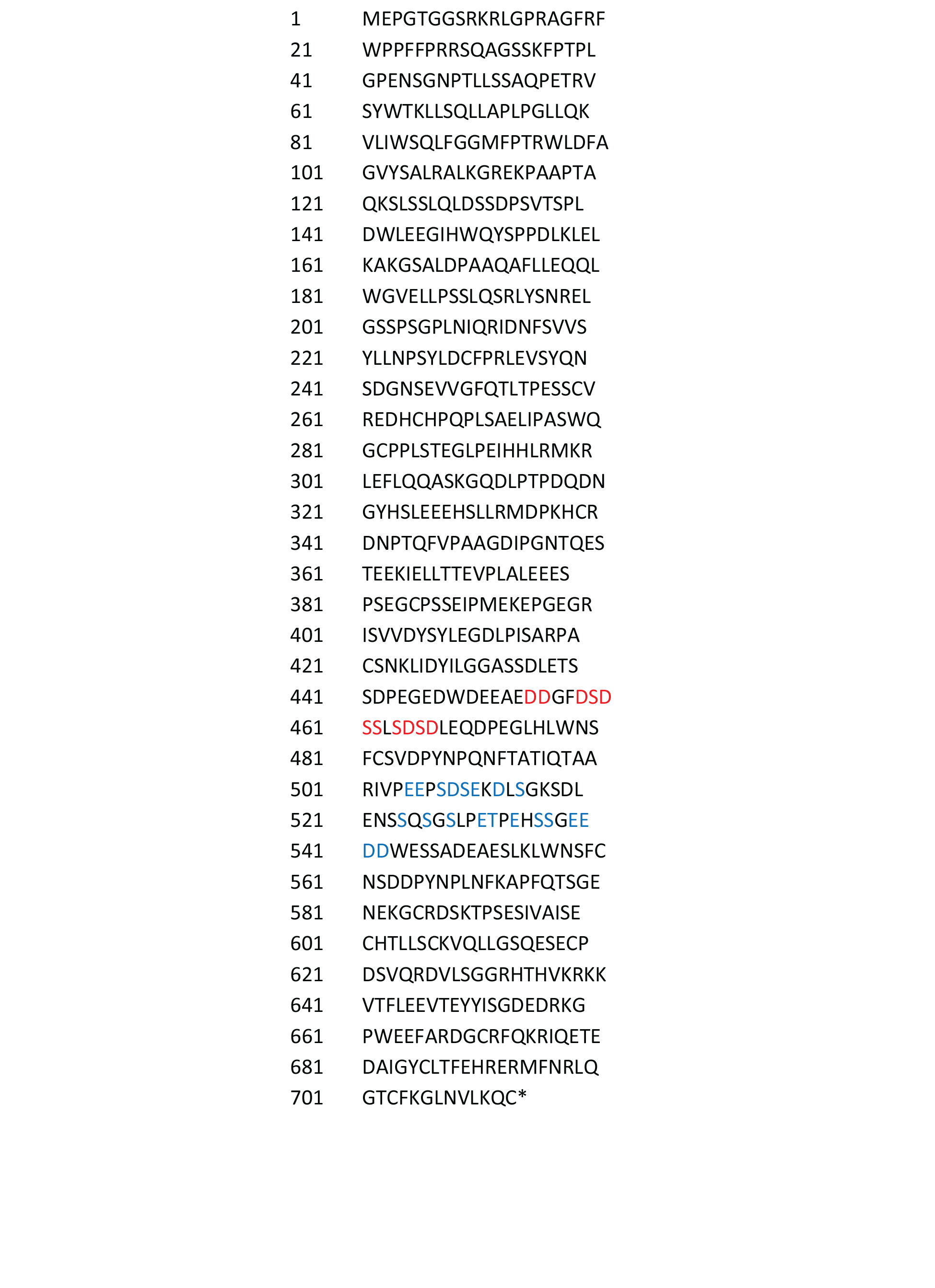

Supplement: S10 Fig — Residues mutated to alanine in the 11A mutant are marked in red. The 31A mutant includes those alanines as well as alanines in place of the residues marked in blue. (TIF) [file pgen.1005292.s010.tif]

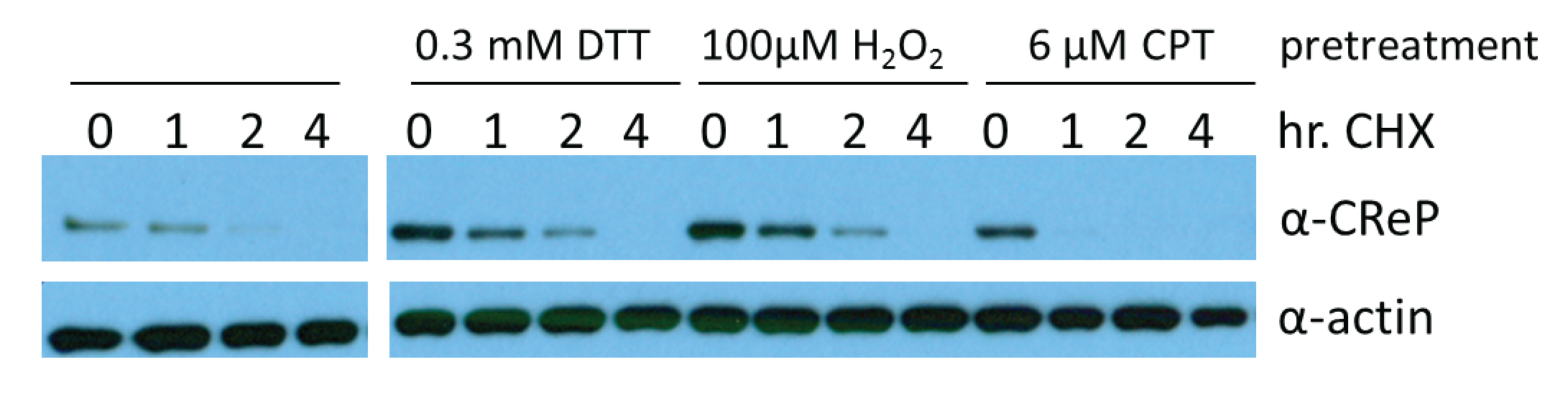

Supplement: S11 Fig — Cells were treated with the indicated concentrations of the indicated drugs for 2.5 hours before addition of cycloheximide for the indicated time. (TIF) [file pgen.1005292.s011.tif]

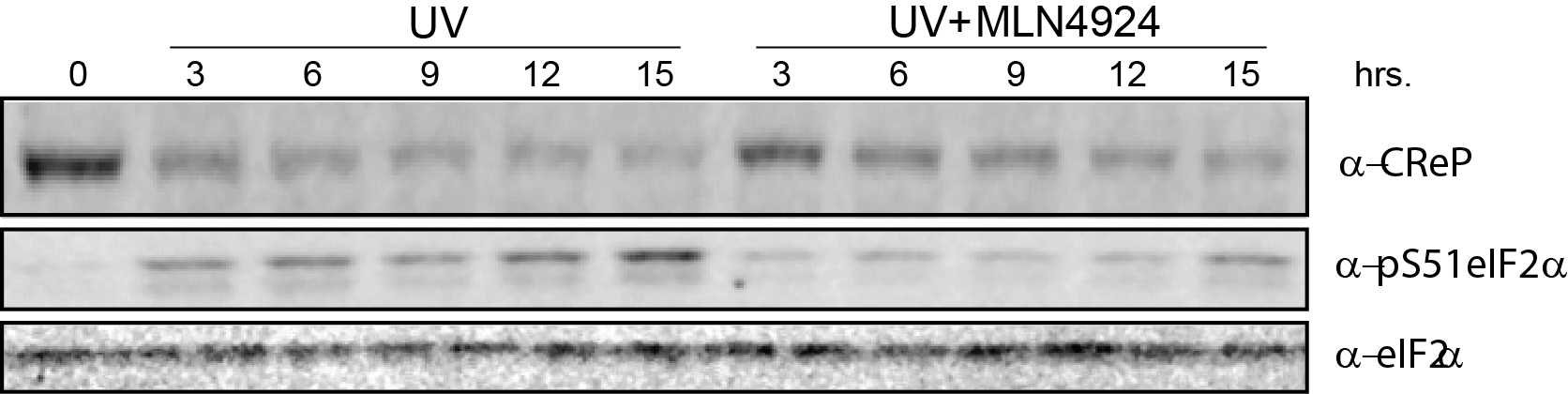

Supplement: S12 Fig — Immortalized MEFs were treated with 300 J/m2 UV-C light for the indicated time, and simultaneously with 1 μM MLN4924 where indicated. (TIF) [file pgen.1005292.s012.tif]
